# Expression and Function Analysis of Interleukin-17A/F1, 2, and 3 Genes in Yellow Catfish (*Pelteobagrus fulvidraco*): Distinct Bioactivity of Recombinant IL-17A/F1, 2, and 3

**DOI:** 10.3389/fimmu.2021.626895

**Published:** 2021-06-29

**Authors:** Xu Zhou, Gui-Rong Zhang, Wei Ji, Ze-Chao Shi, Xu-Fa Ma, Zun-Lan Luo, Kai-Jian Wei

**Affiliations:** ^1^ State Key Laboratory of Environmental Criteria and Risk Assessment, Chinese Research Academy of Environmental Sciences, Beijing, China; ^2^ National Demonstration Center for Experimental Aquaculture Education, Huazhong Agricultural University, Wuhan, China; ^3^ Key Laboratory of Freshwater Animal Breeding, Ministry of Agriculture and Rural Affairs, College of Fisheries, Huazhong Agricultural University, Wuhan, China; ^4^ Key Laboratory of Freshwater Biodiversity Conservation, Ministry of Agriculture and Rural Affairs, Yangtze River Fisheries Research Institute, Chinese Academy of Fishery Sciences, Wuhan, China

**Keywords:** yellow catfish, IL-17A/Fs, expression, bioactivity, phagocytosis, downstream pathway

## Abstract

In mammals, Interleukin-17 cytokine family plays critical roles in both acute and chronic inflammatory responses. In fish species, three Interleukin-17A/F (IL-17A/F) genes have been identified to be homologous to mammalian IL-17A and IL-17F, but little is known about their functional activity. In this study, *Pf*_IL-17A/F1, 2 and 3 genes were cloned from yellow catfish (*Pelteobagrus fulvidraco*) and they differed in protein structure and exon length, implying that they may have divergent bioactivity. Real-time quantitative PCR analyses revealed that three *Pf*_IL-17A/F genes were highly expressed in blood and mucosal tissues (skin+mucus and gill) from healthy adult fish. The mRNA expressions of *Pf*_IL-17A/F1, 2 and 3 genes were significantly up-regulated in the gill, skin+mucus, head kidney and spleen after challenge with *Edwardsiella ictaluri* and in the isolated peripheral blood leucocytes (PBLs) of yellow catfish after stimulation with phytohaemagglutinin (PHA), lipopolysaccharides (LPS), peptidoglycan (PGN) and polyinosinic-polycytidylic acid (Poly I:C). These results indicate that *Pf*_IL-17A/F1, 2 and 3 genes may play a vital role in the regulation of immune against pathogens. Additionally, the recombinant (r) *Pf*_IL-17A/F1, 2 and 3 proteins significantly induced the mRNA expressions of proinflammatory cytokines, chemokines and antibacterial peptides genes, and the r*Pf*_IL-17A/F 2 and 3 proteins promoted phagocytosis of PBLs more powerfully than the r*Pf*_IL-17A/F1. Furthermore, the r*Pf*_IL-17A/F1, 2 and 3 proteins might activate the NF-κB and MAPK signal pathways by IL-17RA, ACT1, TRAF6, TRAF2, TRAF5 and TAK1, indicating that the three *Pf*_IL-17A/F proteins may play different roles in promoting inflammatory response.

## Introduction

Water quality is one of the most critical factors of healthy aquaculture. The deterioration of the water environment can reduce fish growth rate and lower fish immunity, which may lead to the invasion of pathogens and the occurrence of fish diseases. Inflammation is broadly defined as a protective response of the organism to stimulation by invading pathogens or endogenous signals such as damaged cells (1). Moderate inflammation can accelerate the clearance of pathogens and tissue repair. Cytokine, as the medium of communication between cells, plays an important regulative role in the development of inflammation (2). As an important cytokine family, the interleukin-17 family can promote inflammation by activating downstream pathways to induce the expression of antimicrobial peptides, inflammatory cytokines, and chemokines (3).

In mammals, the interleukin-17 (IL-17) cytokine family comprises six members based on the structure similarity: IL-17A, IL-17B, IL-17C, IL-17D, IL-17E, and IL-17F. IL-17A, originally called cytotoxic T-lymphocyte antigen 8 (CTLA8) (4), is a symbolic cytokine that plays a redundant role in inflammation, host defense, and tumorigenesis (5). IL-17A and IL-17F are most similar in amino acid sequences, and they are co-expressed and play overlapping roles in the inflammatory response (6). IL-17A and IL-17F were considered to be released by the activated CD4^+^ T cell. In 2005, IL-17-producing T cells were classified as a new distinct CD4^+^ T cell subset, T helper 17 cells (Th17) (7). In mouse, the naïve CD4^+^ T cells can differentiate into Th17 cells under the control of a set of cytokines including TGF-β, IL-6, IL-1β, IL-23, and IL-21, which lead to the activation of retinoid-related orphan nuclear receptor γt (ROR-γt) and signal transducer and activators of transcription 3 (STAT3) pathways (8–12). IL-2, interferon-γ (IFN-γ), IL-4 or IL-12 on these pathways can suppress the differentiation of Th17 cells (7, 13, 14). Though Th17 cells were thought as a major source of IL-17A and IL-17F, IL-17A and IL-17F can also be produced by T cells (Tc-17, γδ-17, NKT-17), innate lymphoid cells (monocyte, neutrophils, NK), and other non-immune cells (paneth cells, epithelial cells) (15, 16). Upon infection and wounding, IL-17A and IL-17F are rapidly produced by immune and non-immune cells or later by adaptive Th17 cells, subsequently these two released cytokines can induce the expression of host defense genes, such as cytokines (IL-1β, TNF, IL-6, and GM-CSF), chemokines (CXCL1, CXCL2, and CXCL8) and antibacterial peptides (β-defensin and S100 proteins) in targeting cells (epithelial cells, keratinocytes, endothelial cells, fibroblasts, and muscle cells), which will further activate NFκB, MAPKs and C/EBPs downstream pathways to protect the host from harmful pathogens (17, 18). Though the roles of IL-17A and IL-17F overlap, they exist in distinct roles in host defense mechanisms. In the colon, IL-17A was produced mainly in T cells, whereas IL-17F was produced in T cells, immune cells and epithelial cells. Furthermore, IL-17A induced inflammatory cytokines more strongly than IL-17F in the autoimmunity, but IL-17F has more critical roles than IL-17A in protecting colonic epithelial cells against the invasion of bacteria (19, 20).

In teleosts, five members of IL-17 family genes have been identified except IL-17E. Three IL-17A or IL-17F homologous genes have been found in zebrafish (*Danio rerio*) and named IL-17A/F1, IL-17A/F2 and IL-17A/F3, because it is difficult to distinguish IL-17A and IL-17F genes (21). Besides, IL-17A/F1, 2 and 3 genes have been identified in medaka (*Oryzias latipes*), Japanese pufferfish (*Takifugu rubripes*), rainbow trout (*Oncorhynchus mykiss*), Atlantic salmon (*Salmo salar*), channel catfish (*Ictalurus punctatus*), sea bass (*Dicentrarchus labrax*), large yellow croaker (*Larimichthys crocea*) and miiuy croaker (*Miichthys miiuy*) (22–28). Zebrafish IL-17A/F1 and 2 genes are localized on the same chromosome and are different from zebrafish IL-17A/F3 (22). Moreover, fish IL-17A/F1 and 3 were clustered in a branch on the phylogenetic trees, showing that fish IL-17A/F1 and 3 share the greatest structural homology (28). There have been many studies on tissue expression and immunoregulation of teleost IL-17A/F genes. Teleost IL-17A/F genes are mainly expressed in immune-related tissues (e.g. kidney, head kidney, and spleen) and mucosal tissues (e.g. gills, skin, and intestine) (23–28), and their gene expression can be modulated following bacterial infection and *in vitro* or *in vivo* stimulation of lipopolysaccharides (LPS), phytohaemagglutinin (PHA) and other immune stimuli (25–28). However, there have been limited researches on the bioactivity of IL-17A/Fs in teleosts, such as rainbow trout, common carp, grass carp and large yellow croaker. Recombinant rainbow trout IL-17A/F2 can induce the expression of chemokines (IL-6 and CXCL8) and antibacterial peptide (β-defensin3) in splenocytes (29). Common carp rIL-17A/F2a can induce the expression of pro-inflammatory cytokine (IL-1β) and antimicrobial peptides (S100A1, S100A10a, and S100A10b) in the primary kidney (30). Grass carp rIL-17A/F1 can enhance the expression of some pro-inflammatory cytokines genes (IL-1β, TNF, IL-6, and CXCL-8), possibly by activating the NF-kB and MAPKs pathways (31). Large yellow croaker rIL-17A/Fs can promote the expression of pro-inflammatory cytokines (IL-1β, IL-6, and TNF-α), two chemokines (CXCL8 and CXCL13) and antimicrobial peptide (hepcidin), potentially *via* the NF-κB pathway (28). These results illustrate that fish IL-17A/F genes are involved in host defense by inducing cytokines, chemokines and antimicrobial peptide. But so far limited researches have been reported to compare the similarities and differences among three IL-17A/F genes, and the unique roles of IL-17A/F genes in fish immunity are still unclear.

Yellow catfish (*Pelteobagrus fulvidraco*) is an important commercial freshwater fish in China. With the rapid development of the aquaculture industry in recent years, yellow catfish suffers from several kinds of bacterial diseases such as ascites disease, enteric septicemia, and crack-head disease (32, 33). Inflammation can effectively prevent and eliminate invaded pathogens, and IL-17A and F could produce strong inflammatory responses by inducing other pro-inflammatory cytokines, chemokines, and antimicrobial peptides (19, 34). Although three genes of IL-17A/F have been found in various fishes, there is no research to compare their distinct function in fish. In this study, we identified three IL-17A/F homologous genes (*Pf*_IL-17A/F1, 2, and 3) in yellow catfish and investigated the mRNA expression of *Pf*_IL-17A/F1, 2 and 3 genes in various tissues. Subsequently, we detected the expression changes of three *Pf*_IL-17A/F genes in gill, skin+mucus, head kidney and spleen after challenge of *Edwardsiella ictaluri*, and analyzed the expression modulation of three *Pf*_IL-17A/F genes in isolated peripheral blood leucocytes (PBLs) of yellow catfish after stimulation with PHA, LPS, PGN and Poly I:C. Moreover, recombinant *Pf*_IL-17A/F1, 2 and 3 proteins were produced and used to compare their bioactivity in PBLs to modulate the mRNA expression of proinflammatory cytokines, chemokines and antimicrobial peptides genes, promote phagocytosis, and activate the downstream signal pathways. The results will provide useful information to better understand the expression patterns and potential functions of three IL-17A/F genes against pathogenic microbes in teleosts.

## Materials and Methods

### Fish Collection

Healthy adult yellow catfish (one year old, ~50 g) were collected from the Liangzi Lake, Hubei Province, and were transported to the fish breeding base of Huazhong Agricultural University (HZAU). Before artificial reproduction, gene cloning and mRNA tissue distribution experiments, the fishes were acclimatized to laboratory conditions in two circulating water tanks (water temperature at ~26°C, dissolved oxygen at ~6.5 mg/L, and pH at ~8.5) and were fed a commercial diet (Hubei Haid Feeds Company, Wuhan, China) twice a day (09:00 and 16:00). The offsprings from artificial reproduction were cultivated in the circulating water tanks to provide fish samples for bacterial challenge and isolation of PBLs.

### Cloning of cDNA Sequences for Three Genes

Three gene-specific primer pairs (IL-17A/F1-F1/IL-17A/F1-R1, IL-17A/F2-F1/IL-17A/F2-R1, and IL-17A/F3-F1/IL-17A/F3-R1) and three 3’-untranslated region (UTR)-specific primers were designed based on the previous transcriptome data of yellow catfish to obtain the core cDNA sequences of the *Pf*_IL-17AF1, 2 and 3 genes (**Supplemental Table 1**). The open reading frame (ORF) and 3’ UTR of three *Pf*_IL-17A/F genes were obtained using PCR and 3’ nested rapid amplification of cDNA ends (RACE) PCR, respectively. The PCR product was cloned into the pMD18-T vector (TaKaRa, China) and sequenced as described previously (35).

The completed sequence of the ORF of the target gene was found using the ORF Finder (http://www.ncbi.nlm.nih.gov/projects/gorf/). The theoretical isoelectric point and molecular weight of the amino acid were predicted using the ExPASy (https://web.expasy.org/compute_pi/). The protein structure of the target gene was predicted by the Simple Modular Architecture Research Tool (SMART) (http://smart.embl-heidelberg.de/). Homologous sequences of the *Pf*_IL-17A/F1, *Pf*_IL-17A/F2 and *Pf*_IL-17A/F3 genes were searched in GenBank with the BLAST program (https://blast.ncbi.nlm.nih.gov/Blast.cgi). Amino acid sequence similarity and identity were computed using the Sequence Manipulation Suite (http://www.bio-soft.net/sms/index.html). A neighbor-joining (NJ) phylogenetic tree was constructed for three *Pf*_IL-17A/F genes and their homologues from other vertebrates based on the amino acid sequences using the MEGA 6.06. Protein secondary structure was predicted using Jpred4 program (http://www.compbio.dundee.ac.uk/jpred/). Disulfide bonding and cysteine connectivity were predicted using the DISULFIND program (http://disulfind.dsi.unifi.it/). The synteny of IL-17A/Fs loci was analyzed using Genomicus (https://www.genomicus.biologie.ens.fr/genomicus-91.01/cgi-bin/search.pl).

### Tissue Distribution

Fifteen adult yellow catfish (~50 g) were anesthetized with tricaine methanesulfonate (MS-222, 300 mg/L), and 15 different tissues, including blood, swim bladder, skin+mucus, hindgut, liver, stomach, brain, gill, spleen, head kidney, muscle, heart, testis, fin and trunk kidney, were rapidly collected for RNA isolation. Every five fishes were as a group of biological duplication. All tissues were immediately frozen in liquid nitrogen and stored at –80°C until RNA extraction.

### Modulation of *Pf*_IL-17AF1, 2 and 3 Gene Expression *In Vivo* and *In Vitro*

To examine the immune responses of the *Pf*_IL-17A/F1, 2 and 3 genes after bacterial challenge, juvenile individuals of yellow catfish (~14 g) were collected at the fish breeding base of HZAU. The bacteria (*E. ictaluri*) for immune challenge experiments were obtained from the fish immunology laboratory of HZAU, and were cultured on brain-heart infusion (BHI) and incubated 12 h at 28°C in the incubator. Fish were injected intraperitoneally with 200 μL of suspended *E. ictaluri* in phosphate-buffered saline (PBS, pH 7.2) with a concentration of 1.5×10^7^ CFU/mL as the experimental group or with the same volume of PBS as the control group. Fifteen fish were randomly sampled from the experimental group at 3, 6, 12, 24, 48, 72 and 120 hours post-injection and from the control group at 0 hours, and every five fishes at each time point were as a group of biological duplication. The sampled fish were anesthetized with 300 mg/L MS-222, and then the gill, skin+mucus, spleen and head kidney tissues were collected for RNA extraction. All tissues were immediately frozen in liquid nitrogen and stored at –80°C until RNA extraction.

To examine the immune responses of the *Pf*_IL-17A/F1, 2 and 3 genes in isolated peripheral blood leucocytes (PBLs) of yellow catfish after stimulation with LPS, PHA and Poly I:C, the blood sample was collected from adult individuals of yellow catfish (~35 g), and PBLs were isolated by density gradient centrifugation as described (35). 5×10^6^ cells were incubated in RPMI (RPMI with L-glutamine, penicillin, streptomycin and 20% fetal bovine serum (FBS); Solarbio, Beijing, China) with 30 μg/mL PHA (Yuanye, Shanghai, China), 15 μg/mL LPS (Sigma, USA), 15 μg/mL PGN (Ryon, Shanghai, China), and 15 μg/mL Poly I:C (Ryon, Shanghai, China), respectively. The cells were gathered by centrifugation at 3, 6, 12 and 24 h after treatment with PHA, LPS, PGN and Poly I:C. Non-treated cells were used as the negative control. All cells were immediately frozen in liquid nitrogen and stored at –80°C until RNA extraction.

### Production and Purification of r*Pf*_IL-17A/F1, r*Pf*_IL-17A/F2 and r*Pf*_IL-17A/F3 Proteins

The ORF sequence of *Pf*_IL-17A/F1, *Pf*_IL-17A/F2 and *Pf*_IL-17A/F3 genes after removal of the signal peptide sequence was amplified with gene-specific primers (**Table 1**) for prokaryotic expression. After digested with *Sal I* and *EcoR I*, the amplicon was inserted into the multiple cloning sites (MCS) of the pET-32a vector. The recombinant plasmid pET-32a-IL-17A/F1, pET-32a-IL-17A/F2 and pET-32a-IL-17A/F3 were then transformed into *E. coli* BL21 (DE3) competent cells, respectively, and the recombinant IL-17A/F (rIL-17A/F) expression was induced at 37°C for 4 h with IPTG at a final concentration of 1.0 mM. Subsequently, the r*Pf*_IL-17A/F protein was purified by Ni-NTA (nitrilotriacetic acid) affinity chromatography (Sangon, Shanghai, China) according to the manufacturer’s instructions. The identity of the recombinant protein was confirmed by SDS-PAGE as a band with the correct molecular weight. The purified protein was quantitated using the Bradford protein quantitation assay by Nanodrop 2000 (Thermo Electron Corporation, USA).

**Table 1:** Transcriptional fold changes of inflammatory cytokines, chemokines, antibacterial peptides and downstream signaling-related genes in yellow catfish PBLs after treatment with the r*Pf*_IL-17A/F1, 2 and 3 proteins (500 ng/mL) for 3 h, 6 h and 12 h.

Gene category	Gene name	r*Pf*_IL-17A/F1			r*Pf*_IL-17A/F2			r*Pf*_IL-17A/F3		
		3 h	6 h	12 h	3 h	6 h	12 h	3 h	6 h	12 h
Inflammatory cytokine	IL-1β	1.39**	1.86**	1.32	3.69**	3.55**	0.70	142.39**	122.19**	58.63**
	IL-6	2.83*	1.55	1.75	1.71*	2.40**	1.85**	4.35**	4.84**	3.69*
	IL-11	1.09	0.79	1.24	1.47	3.64*	3.21*	9.16**	11.94**	5.81**
	IL-22	1.29	0.55	0.78	1.62	4.15**	1.44	4.31*	0.86	0.30
	TNFα	1.16	1.75*	0.99	2.50*	1.58*	0.75	7.08**	3.95**	1.25
	IFNγ1	0.85	0.83	0.60*	1.13	1.10	1.33*	0.75*	0.78**	0.63**
Chemokine	CXCL1	1.95*	1.26	1.52*	0.79	1.23	1.16	1.02	1.35	1.67*
	CXCL8	2.43*	2.00*	24.79**	2.76*	2.23**	1.35	0.50**	0.46*	2.11
	CXCL11	5.46**	8.79**	2.03**	1.51*	1.46*	1.85**	2.68**	4.40**	8.95**
	CCL3	1.72*	1.24	0.90	1.36	1.04	0.77	6.92**	0.84	0.82
	CCL4	0.73	1.76*	2.09*	0.80	1.69*	2.09**	1.90**	9.91**	3.02*
Antibacterial peptide	S100A	2.95**	1.90**	0.37**	3.04*	2.76**	0.85	2.55**	1.19	0.61*
	LEAP	2.44*	1.05	0.29*	0.11**	3.53*	0.16*	1.38	4.61*	0.10*
	β-defensins	1.51	0.97	0.74	0.22*	4.23*	0.71	2.47*	2.16*	0.54**
Downstream signaling-related gene	IL-17RA	1.72	3.50**	0.12**	0.82	1.24	0.86	3.75*	2.77**	0.79
	ACT1	1.26	4.88**	0.09**	4.21*	1.05	0.52	3.13*	2.41*	0.10**
	TRAF6	3.54*	1.72*	0.19**	1.49	1.44	0.56**	6.07*	6.08**	0.45**
	TRAF2	1.22	4.94**	0.09**	0.79	2.90*	0.21**	5.07*	5.70**	0.48**
	TRAF5	3.44*	2.34*	4.08**	1.10	2.42*	0.37*	24.53**	40.63**	1.52
	TAK1	1.71	1.75*	0.61	1.32	2.00*	0.41**	4.78**	8.58**	1.04

Data are the average transcriptional fold changes of 20 genes at each time point after treatment with three rPf_IL-17A/Fs, which were calculated compared to the control group at the same time point (*controls = 1, n = 3*). The PBLs were isolated from five individuals of yellow catfish and were mixed equally, and the pooled sample was further divided into 12 experiment units for different experimental treatments. Significant difference at each time point compared to the control is indicated by asterisks (*P < 0.05, **P < 0.01).

### Modulation of Gene Expression in Yellow Catfish PBLs by Recombinant IL-17A/F Isoforms

The blood sample was collected from adult individuals of yellow catfish (~35 g), and PBLs were isolated by density gradient centrifugation as described (35). 5×10^6^ cells were incubated in RPMI1640 (RPMI1640 with L-glutamine, penicillin, streptomycin, and 10% fetal bovine serum (FBS); Solarbio, Beijing, China) with different doses of r*Pf*_IL-17A/F1, 2 and 3 proteins (5, 50, and 500 ng/mL) for 6 h, and then the mRNA expressions of IL-1β and CXCL8 genes were detected to compare their changes after different treatments and to screen an appropriate dose for bioactivity analysis of three r*Pf*_IL-17A/F proteins in the yellow catfish PBLs. In the bioactivity experiment of three r*Pf*_IL-17A/F proteins, a dose of 500 ng/mL was used for each r*Pf*_IL-17A/F because the IL-1β and CXCL8 genes had good responses in the yellow catfish PBLs at this treatment dose. Freshly prepared PBLs were stimulated with r*Pf*_IL-17A/F1, 2 and 3 proteins (500 ng/mL) for 3 h, 6 h and 12 h. Medium-treated cells were used as a negative control. Also, the PBLs were treated by r*Pf*_IL-17A/F1, 2 and 3 proteins (500 ng/mL) or NF-κB inhibitor PDTC (0.5 μM, Sigma-Aldrich) and p38 MAPK inhibitor SB203580 (20 μM, Calbiochem, San Diego, USA) for 6 h. All cells were immediately frozen in liquid nitrogen and stored at -80°C until RNA extraction. Afterwards, the mRNA expressions of proinflammatory cytokines (IL-1β, TNF-α, IL-6, IL-11, IL-22, and IFN-γ), chemokine (CXCL1, CXCL8, CXCL11, CCL3, and CCL4), antimicrobial peptide (β-defensin, S100A1, and LEAP) and IL-17A/Fs downstream pathway genes (IL-17RA, ACT1, TRAF6, TRAF5, TRAF2, and TAK1) in the PBLs were examined by qPCR to detect the effects of three recombinant *Pf*_IL-17A/F proteins (**Supplemental Table 1**).

### Phagocytic Assay

PBLs in complete cell culture medium prepared above (2 × 10^6^ cells/mL) were added to 6-well suspension cell culture plates (Corning) and incubated at 28°C with 5% CO_2_. The fresh PBLs were stimulated with r*Pf*_IL-17A/F1, r*Pf*_IL-17A/F2, r*Pf*_IL-17A/F3 proteins (500 ng/mL), and also treated by medium alone as a control. Fluorescent latex beads (Fluoresbrite Yellow Green Microspheres, 1.0 µm in diameter; Polysciences) were added 18 h later at a cell/bead ratio of 1:25, and then the PBLs were incubated for a further 2 h. The cells were harvested using PBS and the supernatant was removed by centrifugation at 400 g for 3 min. Non-ingested beads were removed by centrifugation (at 100 g for 10 min at 4°C) over a 3% BSA and 4.5% d-glucose buffer solution prepared with PBS. The cells were washed with PBS and analyzed with Beckman cytoflex Flow Cytometer, measuring at least 50,000 cells after live cell gating according to the forward scatter (FSC)/side scatter (SSC).

### RNA Extraction and cDNA Synthesis

Total RNA was extracted from various tissues and cells using Trizol Reagent (Invitrogen, USA) according to the manufacturer’s instruction. The quality of total RNA was checked by 1% agarose gel electrophoresis. The concentration of total RNA was determined using a Nanodrop ND-2000 spectrophotometer (Thermo Electron Corporation, USA). The first-strand cDNA was generated using the Revert Aid™ M-MLV Reverse Transcriptase Kit (Promega, USA) following the manufacturer’s instructions. The cDNA products were stored at –20°C.

### qPCR

qPCR was used to detect mRNA expression levels of *Pf*_IL-17A/F1, *Pf*_IL-17A/F2 and *Pf*_IL-17A/F3 genes in various tissues and cells of yellow catfish using a 7300 RT-PCR system (Applied Biosystems, USA). The gene-specific primer pairs for qPCR were designed based on the cloned cDNA sequences of *Pf*_IL-17A/F1, *Pf*_IL-17A/F2 and *Pf*_IL-17A/F3 genes (**Supplemental Table 1**). The β-actin gene (XM_027148463.1) was used as an internal control gene. The PCR reaction mixture consisted of 10 μL LightCycler^®^ 480 SYBR Green I Master (Roche, Germany), 7 μL ddH_2_O, 2 mL cDNA (5 times dilution of a template) and 0.5 μL of either gene-specific primer (10 μM) in a total volume of 20 μL. The qPCR of each sample was performed in triplicate according to the following conditions: 95°C for 10 min, followed by 40 cycles at 95°C for 15 s, annealing temperature for 30 s and 72°C for 30 s respectively. At the end of each PCR reaction, amplification curve and melting curve analyses were performed to check the integrity of the reaction and the quality of the product, respectively. To compare mRNA expressions of *Pf*_IL-17A/F1, *Pf*_IL-17A/F2 and *Pf*_IL-17A/F3 genes, the 2^-ΔΔCt^ method (36) was adopted to calculate the relative expression levels of the target genes.

### Statistical Analyses

All data from the qPCRs were expressed as the mean ± standard error (SEM). One-way analysis of variance (ANOVA) and Duncan’s *post hoc* test (*α* = 0.05) were used to examine the differences of mRNA expression levels among different treatments using STATISTICA 8.0 software. A *t*-test was performed to examine the differences of mRNA expression levels between the control group and the experimental group after different treatments (*α* = 0.05, 0.01). All histograms were plotted using GraphPad Prism 5.0 software.

## Results

### Characterization of *Pf*_IL-17A/F1, *Pf*_IL-17A/F2 and *Pf*_IL-17A/F3 cDNA Sequences

The partial cDNA sequences of the *Pf*_IL-17A/F1, *Pf*_IL-17A/F2 and *Pf*_IL-17A/F3 genes were cloned from yellow catfish. The *Pf*_IL-17A/F1 cDNA consisted of an ORF of 483 bp and a 3’-untranslated region (UTR) of 236 bp. The ORF encoded a protein of 161 amino acids (aa) with a predicted signal peptide (1–27 aa), a typical IL-17 superfamily domain (74–153 aa) and one N-glycosylation site (NDS^73-75^). The 3’-UTR contained three mRNA instability motifs (ATTTA) (**Supplemental Figure 1A**). The estimated theoretical isoelectric point (IP) and molecular weight (MW) of *Pf*_IL-17A/F1 protein were 5.18 and 18.42 kDa, respectively. The *Pf*_IL-17A/F2 cDNA was 816 bp in length, including an ORF of 420 bp and a 3’-UTR of 396 bp. The ORF encoded a protein of 139 aa with a predicted signal peptide (1–20 aa), IL-17 superfamily domain (55–132 aa), and one N-glycosylation site (NRS^55-57^). The 3’-UTR contained two mRNA instability motifs (ATTTA) and three putative polyadenylation signal sequences (AATAAA) (**Supplemental Figure 1B**). The estimated theoretical IP and MW of *Pf*_IL-17A/F2 protein were 5.2 and 15.81 kDa, respectively. The *Pf*_IL-17A/F3 cDNA contained an ORF of 483 bp and a 3’-UTR of 44 bp. The ORF encoded a protein of 160 aa with a predicted signal peptide (1–16 aa) and IL-17 superfamily domain (73–152 aa) (**Supplemental Figure 1C**). The estimated theoretical IP and MW of *Pf*_IL-17A/F3 protein were 5.21 and 18.05 kDa, respectively.

### Sequence and Organization Analysis of *Pf*_IL-17A/F1, *Pf*_IL-17A/F2 and *Pf*_IL-17A/F3 Genes

**Figure 1:**
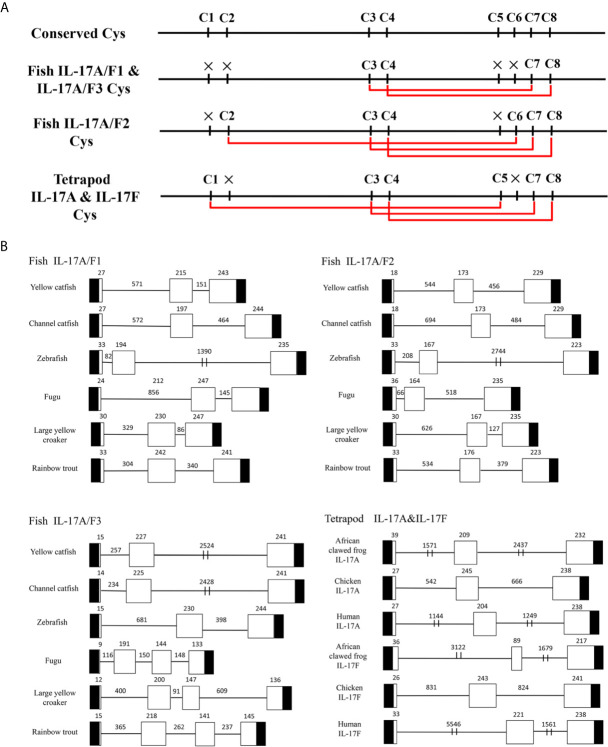
Conserved cysteine residues and predicted potential intra-molecular disulfide bonds in the deduced amino acid sequences of fish IL-17A/F1, 2 and 3 genes and tetrapod IL-17A and F genes **(A)** and comparison of the gene organization of these genes **(B)**. **(A)** Cysteine residues potentially forming disulfide bonds are linked by red lines in tetrapod IL-17A and F, and fish IL-17A/F1, 2 and 3. The disulfide bonds were predicted using DISULFIND. **(B)** The black boxes represent untranslated region, the white boxes represent exons, and the lines represent introns. The genomic sequences are taken from the NCBI database: Yellow catfish, GCF_003724035.1; Channel catfish, GCF_001660625.1; Zebrafish, GCF_000002035.6; Medaka, GCF_002234675.1; Rainbow trout, GCF_002163495; African clawed frog: GCF_001663975.11; Chicken: GCF_000002315.6; human, GCF_000001405.39.

Multiple alignment of amino acid sequences showed that eight cysteine residues were highly conserved in different IL-17A/F genes of vertebrates (**Supplemental Figure 2**). Four conserved cysteine residues of fish IL-17A/F1 and IL-17A/F3 were expected to form two intrachain disulphide bridges (C3/C7 and C4/C8), fish IL-17A/F2 and tetrapod IL-17A and IL-17F possessed six conserved cysteine residues forming three intrachain disulphide bridges, respectively: fish IL-17A/F2 (C2/C6, C3/C7, and C4/C8), tetrapod IL-17A and IL-17F (C1/C5, C3/C7, and C4/C8) (**Figure 1A**). The secondary structures of *Pf*_IL-17A/F1, *Pf*_IL-17A/F2 and *Pf*_IL-17A/F3 proteins all possessed four β-strands as those of human IL-17A and IL-17F, while the length of the fourth β-strand of *Pf*_IL-17A/F2 was shorter than those of *Pf*_IL-17A/F1, *Pf*_IL-17A/F3 and human IL-17A and IL-17F (**Supplemental Figure 2**).

The DNA sequences of *Pf*_IL-17A/F1, *Pf*_IL-17A/F2 and *Pf*_IL-17A/F3 genes all had three exons and two introns as those of other fishes and tetrapod except for the IL-17A/F3 gene organization (four exons and three introns) of fugu, large yellow croaker and rainbow trout (**Figure 1B**). The first exon of IL-17A/F2 in yellow catfish and channel catfish and fish IL-17A/F3 was shorter in length (9–18 bp) than that of other vertebrate IL-17A/F genes (24–39 bp). The second exon of fish IL-17A/F2 (164–176 bp) and African clawed frog IL-17F (89 bp) was very shorter than that of other vertebrate IL-17A/F genes (191–245 bp). The IL-17A/F3 gene of fugu, large yellow croaker and rainbow trout possessed a shorter length of the third exon (144 bp, 147 bp, and 141 bp) and the fourth exon (133 bp, 136 bp, and 145 bp), whereas the IL-17A/F genes of other fishes and tetrapod had a longer length of the third exon (217–247 bp) (**Figure 1B**). Moreover, the introns of IL-17A/F genes were distinctly different in length among various fishes and tetrapod (**Figure 1B**).

### Homology and Phylogenetic Analysis of *Pf*_IL-17A/F1, *Pf*_IL-17A/F2 and *Pf*_IL-17A/F3 Genes

**Figure 2:**
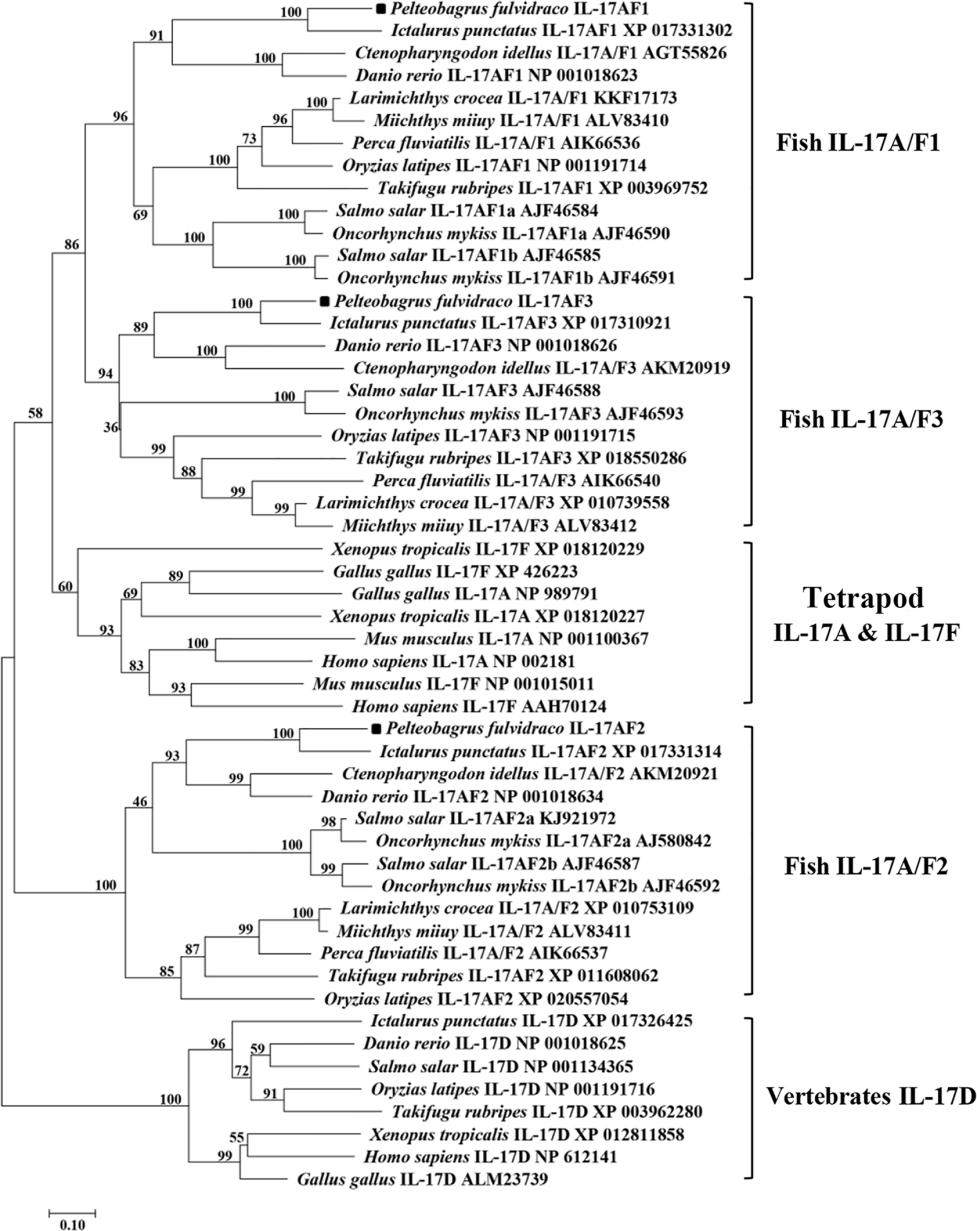
Neighbor-joining (NJ) phylogenetic tree constructed based on deduced amino acid sequences of vertebrate IL-17A/F using MEGA 6.06. A bootstrap analysis is performed using 1000 replicates to test the relative support for particular clades. GenBank accession number for each sequence is given after the species name and molecular type.

Homology analysis of amino acids sequences showed that the *Pf*_IL-17A/F1, *Pf*_IL-17A/F2 and *Pf*_IL-17A/F3 had the highest similarity (69.2%, 79.1%, 83.8%) with the homologues in channel catfish, followed by moderate similarity (51.0%, 55.6%, 57.4%) with zebrafish, and low similarity (44.3%, 41.3%, 42.8%) with medaka. In addition, the three *Pf*_IL-17A/Fs had very lower sequence similarity (27.2%–37.8%) to IL-17A or IL-17F in human, chicken, and western clawed frog (**Supplemental Table 2**). The NJ phylogenetic tree of deduced IL-17A/F amino acid sequences showed that the *Pf*_IL-17A/F1, *Pf*_IL-17A/F2 and *Pf*_IL-17A/F3 of yellow catfish were clustered respectively with those of other teleost IL-17A/Fs into three branches (IL-17A/F1, IL-17A/F2, and IL-17A/F3), of which the IL-17A/F1 and IL-17A/F3 branches were further clustered into a group (**Figure 2**). However the IL-17A and IL-17F of mammals, birds and amphibians were grouped into another branch (**Figure 2**).

### Synteny Analysis of *Pf*_IL-17A/F1, 2, and 3 Genes

**Figure 3:**
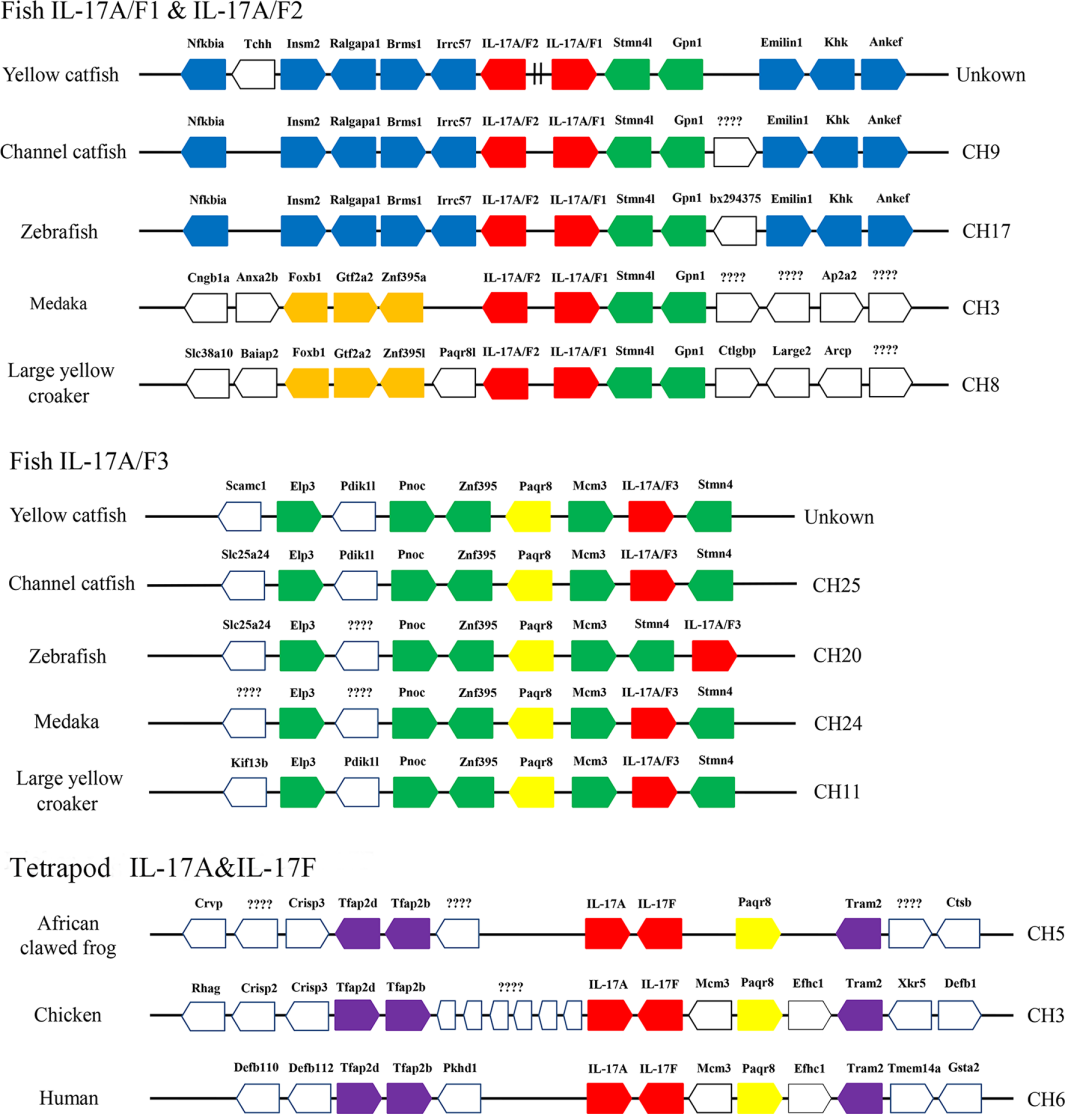
Schematic representation of gene synteny at the IL-17A/Fs loci in teleosts and the IL-17A and IL-17F loci in African clawed frog, chicken and human. The IL-17A/Fs, IL-17A and IL-17F genes are shown in red, the other syntenic genes are shown to be conserved across vertebrates (yellow), only across teleosts (green), only in yellow catfish, channel catfish and zebrafish (blue), only in medaka and large yellow croaker (orange), and only across tetrapod (purple).

Syntenic analysis of IL-17A/F genes showed the synteny arrangements of three IL-17A/F genes were conserved across teleosts, which were different from that of tetrapod IL-17A and IL-17F genes (**Figure 3**). Unlike tetrapod IL-17A and IL-17F genes, fish IL-17A/F1 and IL-17A/F2 genes were adjacent and localized on the same chromosome, whereas fish IL-17A/F3 gene was localized on another chromosome (**Figure 3**). The membrane progestin receptor beta-like (paqr8) gene was conserved across vertebrates. In addition, seven genes (mcm3, stmn4l, gpn1, elp3, pnoc, znf395, and stmn4) were conserved across teleosts, and eight genes (nfkbia, insm2, ralgapa1, brms1, irrc57, emilin1, khk, and ankef) were only conserved across yellow catfish, channel catfish and zebrafish.

### Expression Pattern of *Pf*_IL-17A/F1, 2 and 3 Genes in Various Tissues From Healthy Adults

**Figure 4:**
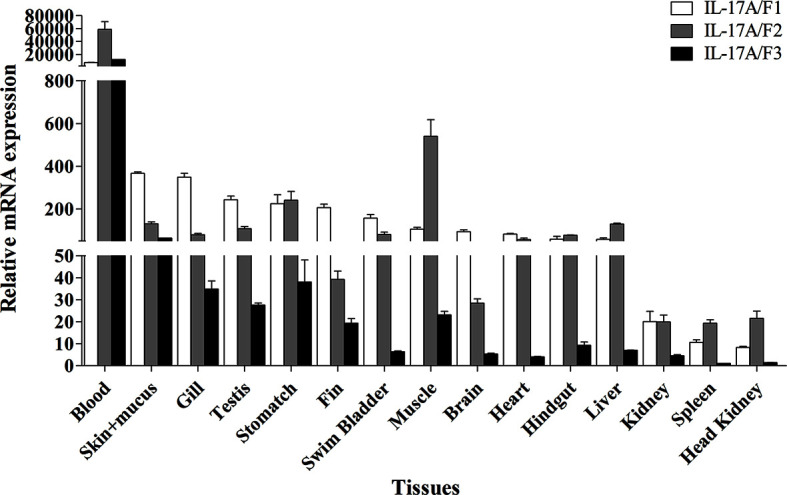
Tissues expression levels of *Pf*_IL-17A/F1, *Pf*_IL-17A/F2 and *Pf*_IL-17A/F3 mRNAs in adult yellow catfish blood, swim bladder, skin+mucus, hindgut, liver, stomach, brain, gill, spleen, head kidney, muscle, heart, testis, fin and trunk kidney. The mRNA expressions of three IL-17A/F genes were detected using qPCR and normalized to the internal gene β-actin. Columns represent the means for each treatment (*n* = 3). Error bars represent standard error of the means.

The tissue distributions of *Pf*_IL-17A/F1, *Pf*_IL-17A/F2 and *Pf*_IL-17A/F3 mRNA expressions were detected by qPCR in various tissues of adult yellow catfish (**Figure 4**). The results showed that *Pf*_IL-17A/F1, 2 and 3 genes were constitutively expressed in all 15 examined tissues, with an extremely high expression level in the blood. *Pf*_IL-17A/F1 and *Pf*_IL-17A/F2 mRNAs were expressed with a high level in the skin+mucus, gill, testis, stomach, swim bladder, muscle, heart, hindgut and liver, and a relatively low level in other tissues. *Pf*_IL-17A/F3 gene was expressed with a high level in the skin+mucus, and a relatively low level in other tissues. Three *Pf*_IL-17A/F genes were expressed at a low level in the kidney, spleen and head kidney. Overall, the expressions of *Pf*_IL-17A/F1 and *Pf*_IL-17A/F2 mRNAs were higher than those of *Pf*_IL-17A/F3 mRNA in almost all tissues (**Figure 4**).

### Expression of *Pf*_IL-17A/F1, 2 and 3 Genes After Challenge of *Edwardsiella ictaluri* and Stimulation With Stimulators

**Figure 5:**
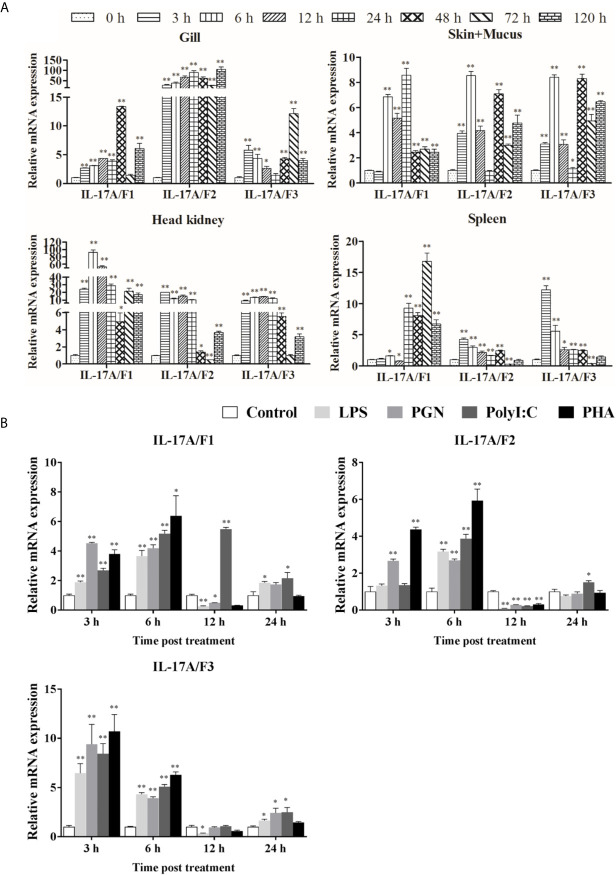
*Pf*_IL-17A/F1, 2 and 3 mRNAs can be induced after the infection of bacteria and the stimulation with stimulators. **(A)** The changes of relative expression levels in the gill, skin+mucus, head kidney and spleen of yellow catfish after challenge of *Edwardsiella ictaluri*. Columns and deviation bars represent the means for each treatment (*n* = 3) and the standard errors of the means, respectively. **(B)** The changes of expression levels in PBLs of yellow catfish after stimulation with LPS, PGN, Poly I:C and PHA. The PBLs were isolated from five individuals of yellow catfish and were mixed equally, and the pooled sample was further divided into 20 experiment units for different experimental treatments. Columns and deviation bars represent the means for each treatment (*n* = 3) and the standard errors of the means, respectively. Significant differences at different time points after challenge compared to the control are indicated by asterisks (*P < 0.05, **P < 0.01).

The expression levels of *Pf*_IL-17A/F1, 2 and 3 mRNAs were detected in the gill, skin+mucus, head kidney and spleen tissues of yellow catfish after challenge with *E. ictaluri* (**Figure 5A**). In the gill, the expression of *Pf*_IL-17A/F1 mRNA was significantly up-regulated from 3 h to 120 h with the peak level at 48 h (*P* < 0.01) except for returning to the control level at 72 h, the expression of *Pf*_IL-17A/F2 mRNA was significantly up-regulated from 3 h to 120 h with the highest value at 120 h (*P* < 0.01), and the expression of *Pf*_IL-17A/F3 mRNA was significantly up-regulated from 3 h to 120 h with the peak at 72 h except for a decline to the control level at 24 h (**Figure 5A**). In the skin+mucus, the expression of *Pf*_IL-17A/F1 mRNA was significantly up-regulated from 6 h to 120 h and reached the peak at 24 h (*P* < 0.01), and the expression of *Pf*_IL-17A/F2 and *Pf*_IL-17A/F3 mRNAs was significantly up-regulated from 3 h to 120 h with the peak level at 6 h (*P* < 0.01) except that the *Pf*_IL-17A/F2 mRNA expression declined to the control level at 24 h (**Figure 5A**). In the head kidney, the expression of *Pf*_IL-17A/F1 mRNA was significantly up-regulated from 3 h to 120 h with the highest level at 6 h (*P* < 0.01) except for a decline to a moderate level at 48 h (*P* < 0.05), the expression of *Pf*_IL-17A/F2 mRNA was significantly up-regulated from 3 h and 120 h (*P* < 0.01 or 0.05) except for a significant down-regulation at 72 h (*P* < 0.01), and the expression of *Pf*_IL-17A/F3 mRNA was significantly up-regulated from 3 h and 120 h (*P* < 0.01) except a decrease to the control level at 72 h (**Figure 5A**). In the spleen, the expression of *Pf*_IL-17A/F1 mRNA was significantly up-regulated from 24 h to 120 h with the peak at 72 h (*P* < 0.01) after a fluctuation from 3 h to 12 h, the expression of *Pf*_IL-17A/F2 and 3 mRNAs was significantly up-regulated from 3 h to 48 h with the peak at 3 h (*P* < 0.01 or 0.05), afterwards it was significantly down-regulated to a low level at 72 h (*P* < 0.01) and returned to the control level at 120 h (**Figure 5A**).

To further explore the immunoregulation of *Pf*_IL-17A/F1, 2 and 3 genes, the expressions of *Pf*_IL-17A/F1, *Pf*_IL-17A/F2 and *Pf*_IL-17A/F3 mRNAs in isolated peripheral blood leucocytes (PBLs) of yellow catfish were detected after stimulation with LPS, PGN, poly I:C and PHA (**Figure 5B**). After LPS and PGN stimulation, the expression of *Pf*_IL-17A/F1 mRNA was significantly up-regulated at 3 h and 6 h (*P* < 0.01), and then it was significantly down-regulated at 12 h (*P* < 0.01 or 0.05) and returned to a moderate level at 24 h, while the expression of *Pf*_IL-17A/F1 mRNA was significantly up-regulated from 3 h to 24 h with the peak at 12 h after poly I:C stimulation (**Figure 5B**). The expression of *Pf*_IL-17A/F2 mRNA was significantly up-regulated at 3 h and 6 h, and it was significantly down-regulated at 12 h after PGN stimulation (*P* < 0.01); while the expression of *Pf*_IL-17A/F1 mRNA was only significantly up-regulated at 6 h and the *Pf*_IL-17A/F3 mRNA was significantly up-regulated at 6 h and 24 h, and they were significantly down-regulated at 12 h after LPS and Poly I:C stimulation (*P* < 0.01) (**Figure 5B**). After LPS, PGN and Poly I:C stimulation, the expression levels of *Pf*_IL-17A/F1, 2 and 3 mRNAs were significantly up-regulated at 3 h, 6 h and 24 h with the highest values at 3 h, and they decreased to a level lower or close to the control at 12 h (**Figure 5B**). After PHA stimulation, the expression levels of *Pf*_IL-17A/F1 and 2 mRNAs were up-regulated at 3 h and 6 h with the peak value at 6 h (*P* < 0.01 or 0.05), subsequently they were significantly down-regulated at 12 h (*P* < 0.01) and increased to the control level at 24 h, while the expression of *Pf*_IL-17A/F3 mRNA was up-regulated at 3 h and 6 h with the peak at 3 h (*P* < 0.01) and decreased to a level close to the control at 12 h and 24 h (**Figure 5B**).

### Production, Purification, and Dose Screening of Three Recombinant *Pf*_IL-17A/F Proteins

**Figure 6:**
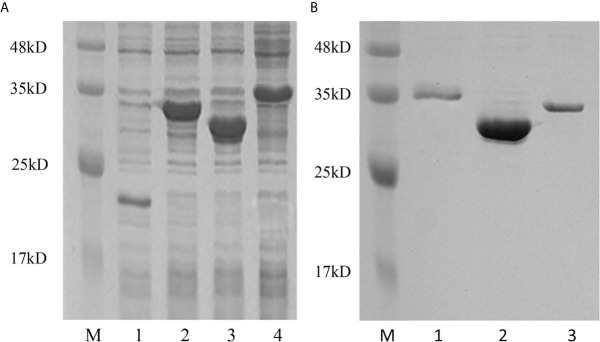
Production and purification of recombinant *Pf*_IL-17A/F1, 2 and 3 proteins by SDS-PAGE. **(A)** Production of r*Pf*_IL-17A/F1, 2 and 3 proteins. Lane 1: Tag protein; Lane 2: r*Pf*_IL-17A/F1 protein; Lane 3: r*Pf*_IL-17A/F2 protein; Lane 4: r*Pf*_IL-17A/F3 protein. **(B)** Purification of three r*Pf*_IL-17A/Fs proteins. Lane 1: r*Pf*_IL-17A/F3 protein; Lane 2: r*Pf*_IL-17A/F2 protein; Lane 3: r*Pf*_IL-17A/F1 protein.

**Figure 7:**
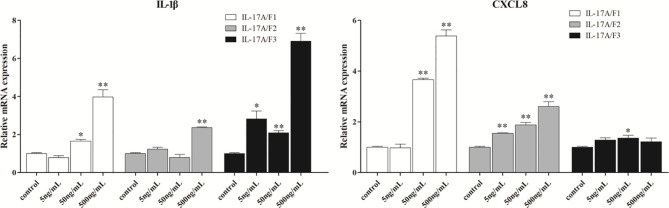
Effects of recombinant *Pf*_IL-17A/F1, 2 and 3 dose course on the expressions of IL-1β and CXCL8 mRNAs in PBLs of yellow catfish. PBLs were treated with different doses of r*Pf*_IL-17A/F1, 2 and 3 proteins (5, 50, 500 ng/mL) for 6 h. Transcriptional fold changes of the IL-1β and CXCL8 genes at different treatment doses were calculated compared to the control group. The PBLs were isolated from four individuals of yellow catfish and were mixed equally, and the pooled sample was further divided into 12 experiment units for different experimental treatments. Columns and deviation bars represent the means and standard errors for each treatment (*n* = 3), respectively. Significant difference at different treatment doses compared to the control is indicated by asterisks (*P < 0.05, **P < 0.01).

To determine the biological function of *Pf*_IL-17A/F 1, 2 and 3, three recombinant (r) *Pf*_IL-17A/F proteins were expressed as an N-terminal 6-histidine-tagged fusion protein in *E. coli* and purified with nickel-nitrilotriacetic acid (Ni-NTA) metal affinity chromatography. SDS-PAGE analysis showed that the molecular mass of r*Pf*_IL-17A/F1, 2 and 3 proteins including His-tag was roughly 33.6, 30.67 and 34.3 kDa, respectively (**Figure 6**). Yellow catfish PBLs were treated with the different concentrations (5, 50, and 500 ng/mL) of r*Pf*_IL-17A/F1, 2 and 3 proteins for 6 h. The dose-course experiments showed that the mRNA expression of IL-1β gene was significantly induced at 500 ng/mL of three r*Pf*_IL-17A/F proteins (*P* < 0.01), and the mRNA expression of CXCL8 gene was notably induced at 500 ng/mL of r*Pf*_IL-17A/F1 and 2 proteins (*P* < 0.01) (**Figure 7**). Therefore yellow catfish PBLs were sensitive to the r*Pf*_IL-17A/F1, 2 and 3 proteins at a dose of 500 ng/mL.

### Modulation of Inflammatory Cytokines, Chemokines, Antimicrobial Peptides and Downstream Signaling-Related Genes’ Expression by Three r*Pf*_IL-17A/F Proteins in Yellow Catfish PBLs

After yellow catfish PBLs were stimulated with the r*Pf*_IL-17A/F1, 2 and 3 proteins (500 ng/mL) for 3 h, 6 h and 12 h, the mRNA expression of inflammatory cytokines, chemokines, antimicrobial peptides and downstream signaling-related genes were detected by qPCR (**Table 1**). IL-1β, TNFα and IL-6 mRNAs were notably induced in yellow catfish PBLs by the r*Pf*_IL-17A/F1, 2 and 3 proteins at some time points (*P* < 0.01 or *P* < 0.05). IL-11 and IL-22 mRNAs were significantly induced in the PBLs by the r*Pf*_IL-17A/F2 and 3 proteins at some time points (*P* < 0.01 or *P* < 0.05). Interestingly, the mRNA expression of IFNγ1 was notably inhibited in the PBLs by the r*Pf*_IL-17A/F1 and 3 proteins at some time points (*P* < 0.05 or 0.01), whereas it was significantly induced by the r*Pf*_IL-17A/F1 protein at 12 h in the PBLs (*P* < 0.05). CXCL11 and CCL4 mRNAs were significantly induced in the PBLs by the r*Pf*_IL-17A/F1, 2 and 3 proteins at some time points (*P* < 0.01 or *P* < 0.05). CXCL1 and CCL3 mRNAs were notably induced in the PBLs by the r*Pf*_IL-17A/F1 and 3 at some time points (*P* < 0.01 or *P* < 0.05). The expression of CXCL8 mRNA was only distinctly induced in the PBLs by the r*Pf*_IL-17A/F1 and 2 proteins at some time points (*P* < 0.05 or 0.01). S100A1 and LEAP mRNAs were distinctly induced in the PBLs by the r*Pf*_IL-17A/F1, 2 and 3 at some time points (*P* < 0.01 or *P* < 0.05). β-defensins mRNA was distinctly induced in the PBLs by the r*Pf*_IL-17A/F2 and 3 proteins at some time points (*P* < 0.05). The mRNA expressions of IL-17RA, ACT1, TRAF6, TRAF2, TRAF5 and TAK1 were distinctly induced in the PBLs by the r*Pf*_IL-17A/F1 and 3 at some time points (*P* < 0.01 or *P* < 0.05). The r*Pf*_IL-17A/F2 proteins can significantly up-regulated the mRNA expressions of ACT1, TRAF2, TRAF5 and TAK1 at some time points (*P* < 0.05) (**Table 1**). On the whole, the r*Pf*_IL-17A/F2 and 3 proteins could induce the mRNA expressions of pro-inflammatory cytokine genes more powerfully than the r*Pf*_IL-17A/F1 protein, the mRNA expression levels of chemokine genes induced by the r*Pf*_IL-17A/F1 and 3 proteins were higher than those by the r*Pf*_IL-17A/F2 protein, and the r*Pf*_IL-17A/F1, 2 and 3 proteins all could induce the mRNA expression of antimicrobial peptides and downstream signaling-related genes in the PBLs of yellow catfish (**Table 1**).

### Three r*Pf*_IL-17A/F Proteins Promote Phagocytosis of Yellow Catfish PBLs

**Figure 8:**
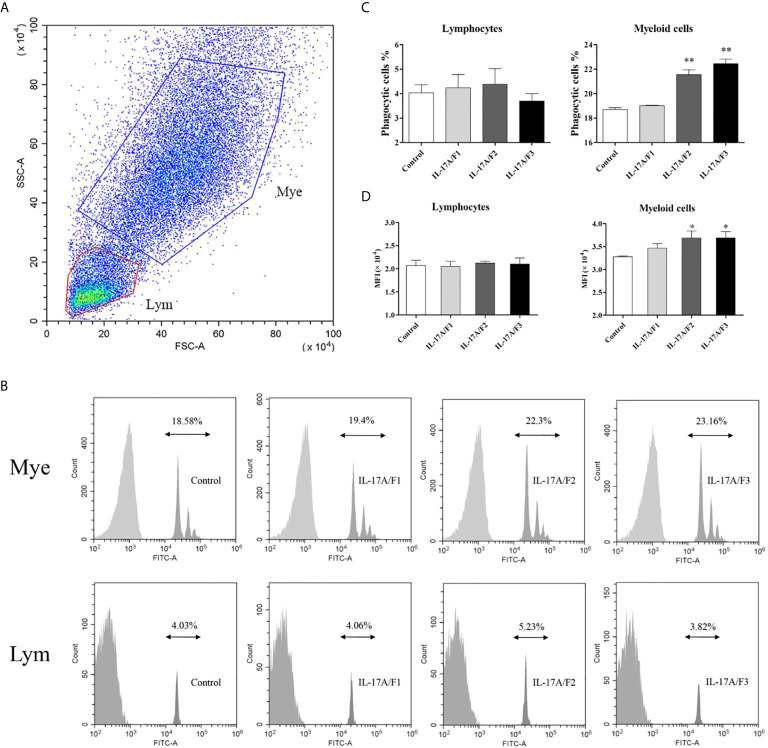
The r*Pf*_IL-17A/F1, 2 and 3 promote phagocytosis of PBLs in yellow catfish. **(A)** Cell typing of PBLs by flow cytometry analysis. Yellow catfish PBLs were incubated with r*Pf_*IL-17A/F1, IL-17A/F2 and IL-17A/F3 proteins (500 ng/mL) or medium alone as a control for 18 h. The PBLs were then incubated with 1.0 µm fluorescent beads for 2 h and analyzed by flow cytometry. Mye: myeloid cells, Lym: lymphocytes. **(B)** The phagocytic activity of PBLs detected by flow cytometry. Typical results from a biological replication experiment are shown. **(C)** The percentage of phagocytic leukocytes in lymphocytes and myeloid cells. **(D)** The mean fluorescence intensity (MFI) of phagocytic cells. The PBLs were isolated from three individuals of yellow catfish and were mixed equally, and the pooled sample was further divided into four experiment units for different experimental treatments. Columns and deviation bars represent the means and standard errors for each treatment (*n* = 3), respectively. Significant difference in different protein treatments compared to the control is indicated by asterisks (*P < 0.05, **P < 0.01).

To investigate the effect of three r*Pf*_IL-17A/F proteins on the phagocytosis of yellow catfish PBLs, the PBLs stimulated with three r*Pf*_IL-17A/F proteins (500 ng/mL) and incubated with fluorescent beads were analyzed by flow cytometry. Lymphocytes (Lym) and myeloid cells (Mye) were gated in the PBLs (**Figure 8A**). The r*Pf*_IL-17A/F2 and 3 proteins significantly promoted the phagocytic activity of myeloid cells in the PBLs (*P* < 0.01), whereas the r*Pf*_IL-17A/F1 protein just slightly increased the phagocytic activity of myeloid cells, and the three r*Pf*_IL-17A/Fs proteins had no obvious effect on the phagocytic activity of lymphocytes in the PBLs (**Figures 8B, C**). Simultaneously, the mean fluorescence intensity (MFI) of myeloid cells was significantly up-regulated in the PBLs after stimulation with the r*Pf*_IL-17A/F2 and 3 proteins (*P* < 0.05), whereas the MFI of lymphoid cells was not altered after three r*Pf*_IL-17A/F proteins treatment (**Figure 8D**).

### Recombinant *Pf*_IL-17A/F1, 2, and 3 Proteins Promoted IL-1β and CXCL11 mRNA Expression in PBLs *via* MAPK and NF-κB Pathway

**Figure 9:**
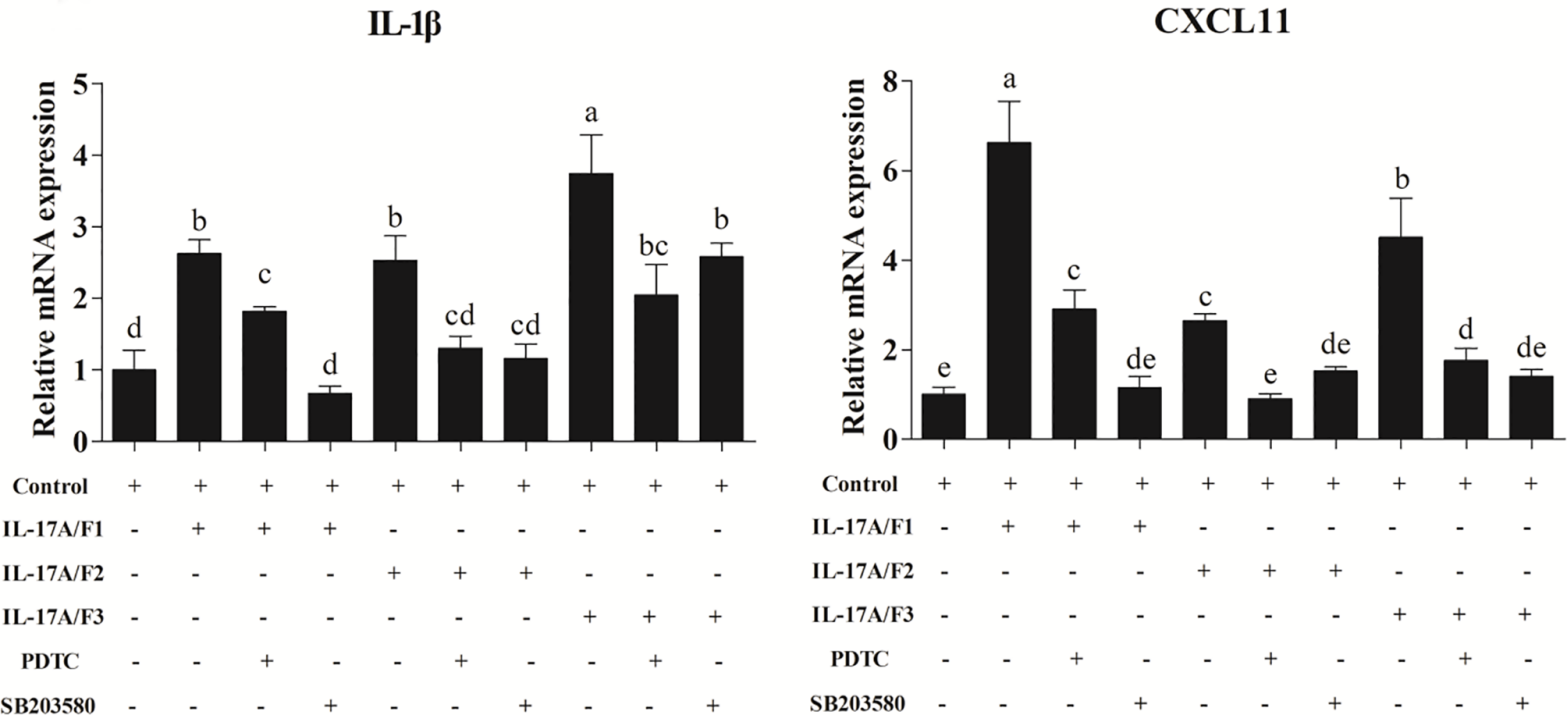
Recombinant *Pf*_IL-17A/F1, 2 and 3 proteins promoted IL-1β and CXCL11 mRNA expression in PBLs *via* MAPK and NF-κB pathway. PBLs were treated with 500 ng/mL r*Pf*_IL-17A/F1, 2 and 3 for 6 h in the presence (+) or absence (-) of different inhibitors (PDTC and SB203580). The mRNA levels of IL-1β and CXCL11 were determined by qPCR. The PBLs were isolated from four individuals of yellow catfish and were mixed equally, and the pooled sample was further divided into ten experiment units for different experimental treatments. Columns represent the means for each treatment (*n* = 3). Error bars represent standard error of the means. Different letters above the bars indicate significant difference among/between the means (One-way ANOVA and Duncan test, *P* < 0.05).

After the yellow catfish PBLs were exposed to the r*Pf*_IL-17A/F1, 2 and 3 (500 ng/mL) for 6 h in the presence of NF-κB inhibitor PDTC (0.5 μM) and p38 MAPK inhibitor SB203580 (20 μM), the IL-1β and CXCL-11 mRNA expressions triggered by the r*Pf*_IL-17A/F1, 2 and 3 were differently blocked by the PDTC and SB203580 (**Figure 9**).

## Discussion

In this study, partial cDNA sequences of three IL-17A/F genes were cloned from yellow catfish. The deduced *Pf*_IL-17A/F1, 2 and 3 amino sequences contained a signal peptide and an IL-17 superfamily domain as the human IL-17A and IL-17F, and their isoelectric points were acidic and close in size (5.18–5.21), indicating that *Pf*_IL-17A/F 1, 2 and 3 genes may have some similar biological functions or signal pathways as the human IL-17A and IL-17F genes (28, 29, 31, 37). Moreover, the *Pf*_IL-17A/F1 and *Pf*_IL-17A/F2 cDNAs possessed several copies of mRNA instability motif (ATTTA), which might regulate the degradation of mRNA in their 3’-UTR (38).

During biological evolution, the two rounds of whole genome duplication (WGD) event dated from 520–550 million year ago in vertebrates (39, 40). Moreover, a teleosts-specific third WGD event happened 226–316 million year ago, and approximately 50% of duplicated genes were retained that may have important roles (41). Therefore, some teleosts may have more homologues in some duplicated genes than mammals. In mammals, IL-17A and IL-17F genes have high amino acid sequence similarity, whereas there have been three or five IL-17A or F homologue genes (IL-17A/F) found in teleosts (22, 26). Fish IL-17A/F1, 2 and 3 had the same genome organization with those of tetrapods (three exons and two introns) except for IL-17A/F3 in fugu, large yellow croaker and rainbow trout (four exons and three introns). In terms of exon length, we inferred that the third exon of IL-17A/F3 in fugu, large yellow croaker and rainbow trout might have been split into two small exons in fish evolution process (22). Mammalian and avian IL-17A and IL-17F are adjacently located on the same chromosomes (22). In fish, IL-17A/F1 and IL-17A/F2 were adjacently located on the same chromosome, but IL-17A/F3 was located on another chromosome, indicating that fish have more IL-17A/F homologue genes than tetrapods because of genome duplication and chromosomal rearrangement in fish biological evolution (22).

Although mammalian IL-17A and IL-17F play important and similar roles in inflammation, IL-17A can induce inflammatory cytokines more strongly than IL-17F in the autoimmunity, and IL-17F plays a more critical role than IL-17A in protecting colonic epithelial cells from bacterial invasion (19, 20). In fish, IL-17A/F1, 2 and 3 are vital pro-inflammatory cytokines through which can promote the mRNA expressions of other pro-inflammatory cytokines, chemokines and antimicrobial peptides, but little is known about the functional difference of fish IL-17A/F1, 2 and 3. Both human IL-17A and IL-17F monomers have two pairs of anti-parallel β-sheets (strands 1–4), with the second and fourth strands connected by two disulfide bonds (6, 42). Similarly, *Pf*_IL-17A/F1, 2 and 3 were predicted to possess four inferred β-strands, the second and fourth strands were linked by two disulfide bonds, while the fourth β-strand of *Pf*_IL-17A/F2 was shorter than those of *Pf*_IL-17A/F1, *Pf*_IL-17A/F3, human IL-17A and IL-17 F. Moreover, *Pf*_IL-17A/F2 also formed another disulfide bond similar to human IL-17A and IL-17F that is not bound by the receptor (42). The IL-17 superfamily domain was encoded by the second and third exons, but the second exon of fish IL-17A/F2 is shorter than those of fish IL-17A/F1 and 3. Furthermore, phylogenetic and homological analysis showed that teleost IL-17A/F1 is more similar to IL-17A/F3 than IL-17A/F2 in amino acid sequence. Different amino acid sequences will construct distinct secondary and tertiary structures and result in functional diversification. These results imply that fish IL-17A/F genes may exist distinct functional diversification due to the impact of their structure difference on receptor-binding affinity/signaling.

In mammals, IL-17A and IL-17F are mainly secreted by Th17 cells (15). Here, the highest expression levels of *Pf*_IL-17A/F1, 2 and 3 mRNAs were detected in blood of healthy yellow catfish. Moreover, PHA, as a stimulator of accelerating T cell proliferation (43), was able to significantly induce the mRNA expressions of *Pf*_IL-17A/F1, 2 and 3 genes in PBLs, indicating that the production of IL-17A/F1, 2 and 3 in yellow catfish is similar to that in mammals by T cells. In mammals, Th17 cytokines can protect the host from pathogens at epithelial and mucosal tissues including the skin, lung and intestine (44). IL-17A is produced by intestinal paneth cells (45). IL-17F mRNA is expressed in colonic epithelial cells (20). Similarly, fish IL-17A/F1, 2 and 3 mRNAs are highly expressed in mucosal tissues (skin, gill, and intestine) and have low expressions in the head kidney in channel catfish, Japanese pufferfish, and large yellow croaker (23, 25, 28). Skin, gill and intestine are all part of the mucosal surface, which is the first barrier exposed to a complicated environment to prevent and eliminate invasive pathogens through recruiting neutrophils and lymphocytes (46). In this study, higher expression levels of *Pf*_IL-17A/F1, 2 and 3 mRNAs were detected in skin+mucus, and low expressions of *Pf*_IL-17A/F1, 2 and 3 mRNAs were present in the kidney, head kidney and spleen. Furthermore, in teleosts, after stimulation of stimulators (LPS or Poly I:C) in PBLs, IL-17A/F1, 2 and 3 mRNAs were notably induced in zebrafish and grass carp (21, 31). After bacterial challenge (*A. hydrophila* or *E. ictaluri*), IL-17A/F1, 2 and 3 transcripts were distinctly up-regulated in the head kidney or mucosal tissues in channel catfish and large yellow croaker (25, 28). Here, the expression levels of *Pf*_IL-17A/F1, 2 and 3 mRNAs were significantly up-regulated in all four detected tissues after challenge of *E. ictaluri* and in yellow catfish PBLs after stimulation of LPS, PGN and Poly I:C. These data suggest that *Pf*_IL-17A/F1, 2 and 3 genes may play a crucial role in the host defense against pathogens infection.

Interestingly, although the expression levels of *Pf*_IL-17A/F1, 2 and 3 mRNAs were low in the head kidney and spleen of healthy yellow catfish, they were rapidly and dramatically up-regulated in the head kidney and spleen after challenge of *E. ictaluri*, indicating that fish IL-17A/F1, 2 and 3 genes need to be modulated strictly like human IL-17A and IL-17F as their persistent and excess expression is harmful for the host (34). In mammals, IL-17A is mainly produced in T cells, whereas IL- 17F is produced in T cells, innate immune cells and epithelial cells (20). The mRNA expression of *Pf*_IL-17A/F3 gene is relatively lower than that of *Pf*_IL-17A/F1 and 2 genes in healthy yellow catfish, but it is more induced after stimulation of stimulators and *E. ictaluri* infection, implying that *Pf*_IL-17A/F1, 2 and 3 genes might have functional diversification.

Although mammalian IL-17A and IL-17F have many overlapping functions, they play different roles in host defense against bacterial infection. IL-17A is more effective to induce pro-inflammatory cytokines than IL-17F (18, 47). In fish, IL-17A/F1, 2 and 3 genes can induce the mRNA expressions of IL-1β, TNFα, IL-6, CXCL8 and antimicrobial peptides (28–31), but little is known about the different roles of IL-17A/F1, 2 and 3 in the immune response.

The pro-inflammatory cytokines can directly activate the immune cells to eliminate invading pathogens. In mammals, IL-1β, TNFα, IL-6, IL-11 and IFNγ can directly activate macrophages and neutrophils (1, 48–50), and IL-22, which is derived from Th17 cells and innate lymphoid cells, acts on epithelial cells (1). In large yellow croaker, three rIL-17A/Fs induced the mRNA expressions of IL-1β, IL-6 and TNF-α in the head kidney (28). In this study, the r*Pf*_IL-17A/F2 and 3 proteins notably induced the mRNA expressions of IL-1β, IL-6, IL-11, IL-22 and TNFα in PBLs at certain time points, while the r*Pf*_IL-17A/F1 protein only significantly induced the mRNA expressions of IL-1β, IL-6 and TNFα at 3 h and/or 6 h. Furthermore, r*Pf*_IL-17A/F2 and 3 proteins enhanced the phagocytosis of myeloid cells except for r*Pf*_IL-17A/F1 protein, whereas three r*Pf*_IL-17A/F proteins were ineffective to promote the phagocytosis of lymphocytes in yellow catfish PBLs. These results indicate that the r*Pf*_IL-17A/F2 and 3 proteins can more significantly induce the expression of pro-inflammation cytokines and contribute to the phagocytosis of myeloid cells compared with the r*Pf*_IL-17A/F1 protein. In mammals, IFNγ, as the symbolic cytokine, can inhibit the development of Th17 cells from naive precursor cells (7). Similarly, the r*Pf*_IL-17A/F2 protein only distinctly up-regulated the mRNA expression of IFNγ in PBLs at 12 h, and the r*Pf*_IL-17A/F1 and 3 proteins notably inhibited the mRNA expression of IFNγ, indicating that fish IL-17A/Fs might control the development of fish T cells whose functions like mammalian Th1 cells.

In mammals, both IL-17A and IL-17F can stimulate the release of CXCL1, CXCL2 and CXCL8, and IL-17A can also recruit and activate neutrophils under certain conditions (3, 47, 51). In grass carp, rIL-17A/F1 enhanced the mRNA expression of CXCL8 in head kidney cells to stimulate PBLs migration (31). Large yellow croaker rIL-17A/Fs could promote the mRNA expressions of CXCL8 and CXCL13 in the head kidney (28). In the present study, three r*Pf*_IL-17A/Fs proteins notably induced the mRNA expressions of CXCL11 and CCL4 in PBLs at 6 h and 12 h, the r*Pf*_IL-17A/F1 and 3 proteins significantly up-regulated the mRNA expressions of CXCL1 and CCL3 at a certain time point, and CXCL8 mRNA was induced by the r*Pf*_IL-17A/F1 and 2 proteins. These results showed that fish IL-17A/Fs can promote the expression of chemokines to recruit and activate immune cells.

In humans, IL-17A can more significantly induce the mRNA expressions of β-defensins-2, S100A7, S100A8 and S100A9 compared with IL-17F in primary human keratinocytes (18). Common carp rIL-17A/F2a could induce the mRNA expressions of S100A1, S100A10a and S100A10b in the primary kidney (30). Rainbow trout IL-17A/F2 promoted the mRNA expression of β-defensins-3 in splenocytes (29). Three large yellow croaker IL-17A/Fs proteins could enhance the mRNA expression of hepcidin in the head kidney (28). In this study, three r*Pf*_IL-17A/F proteins were able to stimulate the mRNA expressions of S100A1 in PBLs at certain time points, and only the r*Pf*_IL-17A/F2 and 3 proteins induced the mRNA expression of β-defensins. Moreover, mammalian LEAP2 is not only highly expressed in hepatocytes, but also is blood-derived antimicrobial peptide and expressed in gastro-intestinal epithelial tissues and monocytes (52, 53). But there is no literature that shows that the expression of the LEAP2 gene is regulated by IL-17 at present. Here, three r*Pf*_IL-17A/F proteins were able to stimulate the mRNA expression of LEAP in PBLs at certain time points. These results uncover that, like mammalian IL-17A and IL-17F, fish IL-17A/Fs can facilitate the expressions of antibacterial peptides to eliminate the invading pathogens.

In mammals, IL-17A and IL-17F combine with IL-17RA and IL-17RC to bind ACT1. Subsequently, ACT1 mediates TRAF6 ubiquitination to activate NF-kB and MAPK pathways, or ACT1 recruits TRAF2 and TRAF5 to enhance mRNA stability (3, 44, 47). In large yellow croaker, three rIL-17A/Fs proteins could activate the NF-kB pathway (28). In yellow catfish, the r*Pf*_IL-17A/F1 and 3 proteins notably induced the mRNA expressions of IL-17RA, ACT1, TRAF6, TRAF2, TRAF5 and TAK1 in PBLs, whereas the r*Pf*_IL-17A/F2 protein only enhanced the mRNA expressions of ACT1, TRAF2, TRAF5 and TAK1 at 3 h or 6 h. Furthermore, the IL-1β and CXCL11 mRNA expressions triggered by the r*Pf*_IL-17A/F1, 2 and 3 were blocked by the NF-κB inhibitor PDTC and the p38 MAPK inhibitor SB203580. These results indicate that compared with mammalian IL-17A and IL-17F, fish IL-17A/F1 and 3 may have similar downstream signal pathways, while the downstream signal pathway of fish IL-17A/F2 may be somewhat different.

The individual variability of fish such as body size and physiological status may have potential effects on immuno-related gene expressions and phagocytosis of PBLs. In this study, to reduce the potential effects of fish individual variability, the randomized block design (3 blocks × *M* experimental treatments) was used to control the test error. The PBLs were isolated from several individuals of yellow catfish and were mixed equally, and the pooled sample was further divided into a certain number of experiment units (*M*) as a block for being dealt with different experimental treatments such as the control and other experiment conditions. The second and the third pooled samples were also divided and treated as above. Therefore, in each experimental treatment (i.e. experimental group), three experiment units were from three different pooled samples; in each block, *M* experiment units were from the same pooled sample, and they are homogeneous. Therefore we used dependent t-test to compare the difference between the control group and each other treatment group, this will help to eliminate or reduce the effect of three pooled samples’ difference on statistical analysis. Additionally, pooling a PBLs sample from several fish individuals might produce leucocyte activation to make graft rejection. However, in this study, the death phenomenon of PBLs was not observed in the pooled PBLs samples during the whole experiment. Even if the pooling produces leucocyte activation, this activation will occur both in the treatment groups by IL-17A/Fs and in the control group without IL-17A/Fs. Through analysis of dependent t-test, the effect of this potential leucocyte activation will be eliminated, and the t-test results will mainly reflect the effect of IL-17A/Fs treatments.

In conclusion, we cloned partial cDNA sequences of three IL-17A/F homologous genes (*Pf*_IL-17A/F1, 2, and 3) from yellow catfish. *Pf*_IL-17A/F1, 2 and 3 genes displayed distinct structural characteristics. *Pf*_IL-17A/F1, 2 and 3 mRNAs were ubiquitously expressed in fifteen examined adult tissues and preferentially expressed in the blood, gill, and skin+mucus. The expressions of *Pf*_IL-17A/F1, 2 and 3 mRNAs were all rapidly and significantly up-regulated in the gill, skin+mucus, head kidney and spleen after *E. ictaluri* challenge and in yellow catfish PBLs after stimulation with PHA, LPS, PGN and poly I:C. Moreover, the r*Pf*_IL-17A/F1, 2 and 3 proteins showed divergent bioactivity in PBLs. The r*Pf*_IL-17A/F1, 2 and 3 proteins significantly induced the mRNA expressions of proinflammatory cytokines, chemokines and antibacterial peptides genes, and the r*Pf*_IL-17A/F 2 and 3 proteins promoted more powerful phagocytosis of PBLs than the r*Pf*_IL-17A/F1 protein. Furthermore, the r*Pf*_IL-17A/F1, 2 and 3 proteins could activate the NF-κB and MAPK signal pathways by IL-17RA, ACT1, TRAF6, TRAF2, TRAF5 and TAK1. The results indicate that *Pf*_IL-17A/F1, 2 and 3 play different roles in promoting the inflammatory response and host defense against bacterial infection. These data will help to better understand the potential function of three IL-17A/F genes in fish immune responses towards pathogen infections.

## Data Availability Statement

The original contributions presented in the study are included in the article/**Supplementary Material**. Further inquiries can be directed to the corresponding authors.

## Ethics Statement

The animal study was reviewed and approved by Institutional Animal Care and Use Committees (IACUC) of HZAU, Wuhan, P. R. China.

## Author Contributions

XZ performed the experiments, analyzed the data, and wrote the manuscript. G-RZ, WJ, X-FM, Z-LL, and K-JW conceived and designed the study. Z-CS helped with the preparation of experimental fishes. XZ, Z-LL, and K-JW revised the manuscript. All authors contributed to the article and approved the submitted version.

## Funding

This study was supported by the Biodiversity Survey and Assessment Project of the Ministry of Ecology and Environment, China (Grant No. 2019HJ2096001006), and the National Natural Science Foundation of China (Grant No. 31772851).

## Conflict of Interest

The authors declare that the research was conducted in the absence of any commercial or financial relationships that could be construed as a potential conflict of interest.

The reviewer (ZL) has declared an institutional affiliation, with no other collaboration, with some of the authors (XZ, G-RZ, WJ, X-FM, K-JW) to the handling editor during the time of review.
