# Supplementary material for: Expression and Function Analysis of Interleukin-17A/F1, 2, and 3 Genes in Yellow Catfish (Pelteobagrus fulvidraco): Distinct Bioactivity of Recombinant IL-17A/F1, 2, and 3
Source: Front Immunol. 2021 Jun 29;12:626895. doi: 10.3389/fimmu.2021.626895 (PMC8276262; doi:10.3389/fimmu.2021.626895)
Supplement: Supplementary file 1 [file DataSheet_1.pdf]

Supplemental materials

Supplemental Figure 1. The nucleotide and putative amino acid sequences of *Pf*_IL-17A/F1 (A), *Pf*_IL-17A/F2 (B) and *Pf*_IL-17A/F3 (C). The start codon and stop codon of the open reading frame are indicated with a wavy line. The signal peptide is underlined. The IL-17 superfamily domain is highlighted in red. The N-glycosylation site is bold and italic. Within 3′-UTR, the putative ATTTA instability motifs are in bold and the polyadenylation signals are in bold and underlined.

A

```
1     ATGGCTTTTAAAACCTCCCTCTTAACGTTACCATATGTGATGATGATGGTGATGATGATGATGATGATGGTCACCCAGGCTGCTCCTCCA
1      M  A  F  K  T  S  L  L  T  L  P  Y  V  M  M  M  V  M  M  M  M  M  M  V  T  Q  A  A  P  P
91    AAGGGAAAATTCCCCGTTCATCATAAAGCTCCAAGCTCTGAAGACGCATCGCCAAAACTCTTCATCCTTGTGGATCTGGAACAGGAAATC
31     K  G  K  F  P  V  H  H  K  A  P  S  S  E  D  A  S  P  K  L  F  I  L  V  D  L  E  Q  E  I
181   AAACCGGCGTCACACTCCATCAGACCCATCCACAATGACTCTATCTCACCCTGGGAGACTAGCTATACGTTTGATTCAAATCGGATTCCG
61     K  P  A  S  H  S  I  R  P  I  H  N  D  S  I  S  P  W  E  T  S  Y  T  F  D  S  N  R  I  P
271   TCTTATCTCCCGGAGGCTCGCTGTCTCCTGAGTGGATGTTTAAACCATGACGGTGTTGAGACGCTGGAGTTGGAGTCCAGACGGATTTTC
91     S  Y  L  P  E  A  R  C  L  L  S  G  C  L  N  H  D  G  V  E  T  L  E  L  E  S  R  R  I  F
361   TGGCAGGTTCCGGTCCTGCAGCGAGTTCGCCGTGGCGATGACAAAAGCTATTACTTCAGACTGGAACATAAAACCATCTCAGTGGGCTGC
121    W  Q  V  P  V  L  Q  R  V  R  R  G  D  D  K  S  Y  Y  F  R  L  E  H  K  T  I  S  V  G  C
451   ACCTGTGTCCGGCCGTACGTGGAGCAGATCTGATGTACTGCAAGATGAAGCGTCTGAGTTCTACTGACTCGCCTTATTATTATTATTTAT
151    T  C  V  R  P  Y  V  E  Q  I  *
571   TAGTTTTCACCTGTTTACCTTTGATGAGATTTTATTTTATTTATTAGTTTCATTAGAAGTTGATGAAGGTTTTTGAGGATGTGTGTTTGC
691   TCTGTTATTTATTCACTGTAGTGATGTTTGAGGAGGAAAAGTGAATTAAACGTATTAATTTATTAAACAAGTCAAAAAAAAAAAAAAA
```

B

```
1     ATGAGCTTAAAGCGAACGTTTGTGTTGTGCTGTGGACTTCTGATGTTGAGTCTCACGCACGCCGCTAACAAACAGACGACGGTCTGTGAC
1      M  S  L  K  R  T  F  V  L  C  C  G  L  L  M  L  S  L  T  H  A  A  N  K  Q  T  T  V  C  D
91    ATCGGTCTGATCATCCCGAAACACTTCCACACGTCAGAGTCAGAGGCGATGGAAGGAAACGGCAACATCAACAACCGCTCTCTGTCTGCC
31     I  G  L  I  I  P  K  H  F  H  T  S  E  S  E  A  M  E  G  N  G  N  I  N  N  R  S  L  S  A
181   TGGAAGTGGATACCTCACGTCAGCACCCACAGGATCCCTAGCGTGATATTTGAGGCAGATTGTGAGCATCATCACTGCACATACCCAAAC
61     W  K  W  I  P  H  V  S  T  H  R  I  P  S  V  I  F  E  A  D  C  E  H  H  H  C  T  Y  P  N
271   ATCCAGCAGCAAAAGGAGCTGAACTCTGTACCTATATACAGCTACATGCTGGTGCTCAAACAGGACCCAAAGAACAGGAAGTGCTTCACT
91     I  Q  Q  Q  K  E  L  N  S  V  P  I  Y  S  Y  M  L  V  L  K  Q  D  P  K  N  R  K  C  F  T
361   GTACATTTCCACAGGGTCACCGTGGGCTGCACGTGTGTGTGGGAAAGATCGTCACCGTGAGAGCGACTTTAACACTTTAACACACACGAA
121    V  H  F  H  R  V  T  V  G  C  T  C  V  W  E  R  S  S  P  *
541   TAAATAAGATGAGATTCATTTAAAAAATGAGACGTTGTTCAATTAAGACGTATGACATTAATTAGTTGTGACAACGTTGTGATTACTTTG
631   TTTGGAACTGTTTGTTATTAAATTGACTTTTGACTGATGTTATACACAGATGTGTCACATCTACACTGTATGTTGTTGTGTATATTTACT
721   GATATACAATAAAATCTACGCACACTATGTTGATTGTAAGATGATGTTAGACGATTTTAGAGAAGATGAGAAATAAAGGTTTATTAGCAA
811   ATCTACTTGTGATAATTGTGAGAAATTGTGAAAATAAAACTATTGATTATTCAAAAAAAAAAAAAAAAAAAAAAAAAA
```

C

```
1     ATGCACATCTCGGTGCTTTTCAAGGTCACATTGTTGTTGGCATTGGGAGTACTGTTCCTCGGAGCAGATCATTCTCCTGCAGGACGGAAA
1      M  H  I  S  V  L  F  K  V  T  L  L  L  A  L  G  V  L  F  L  G  A  D  H  S  P  A  G  R  K
91    GAAGGAAAGAAGGAACGGAAAAGGGGTCCGGCTAAGAAGGGCTCCCAAAGAAAGGCCAGGAAACTACAGCTAACTGTCGATTCTACAATA
31     E  G  K  K  E  R  K  R  G  P  A  K  K  G  S  Q  R  K  A  R  K  L  Q  L  T  V  D  S  T  I
181   GAAAGCCAACTAGATACCTATCAAATATCTCCAAGTCGGTCAATATCACCGTGGACATATGAGGTCTCTTACGACGAATCTCGCATTCCG
61     E  S  Q  L  D  T  Y  Q  I  S  P  S  R  S  I  S  P  W  T  Y  E  V  S  Y  D  E  S  R  I  P
271   AGCCGCATCTTCGAGGCGAAGTGTGAGAAAACAGGCTGCTTGAACAAAGATGGGATTGAAGACGCTGGTCTGGAATCCAAACCCATCTTG
91     S  R  I  F  E  A  K  C  E  K  T  G  C  L  N  K  D  G  I  E  D  A  G  L  E  S  K  P  I  L
361   TACCAATTCCTGGTGCTCAGAAGAGTAAAGGGCAAAAAGAAGGACTATTCCTTCAGGCTGGAGAAACACACCACCAGCGTGGGCTGCACG
121    Y  Q  F  L  V  L  R  R  V  K  G  K  K  K  D  Y  S  F  R  L  E  K  H  T  T  S  V  G  C  T
451   TGTGTTTTACCGAACGTCGTGTCTCACATGTAGTGCTGCTGTTCCTGTGTGTATATGACTGAAAAAAAAAAAAAAAA
151    C  V  L  P  N  V  V  S  H  M  *
```

Supplemental Figure 2. Multiple alignment of the deduced amino acid sequences of *Pf*_IL-17A/F1, 2, and 3 with those of other vertebrates. The amino acid sequences of these genes were predicted using MEGA 6.06. ClustalW program in MEGA 6.06 and BoxShade were used for multiple sequence alignments. Similar amino acid residues are marked as grey shadow and identical residues as black shadow. Absent amino acids are indicated by dashes (-). The eight highly conserved cysteine residues are highlighted in red. Protein secondary structure was predicted using Jpred4 program. →: β-sheet. PF: Yellow catfish; IP: Channel catfish; DR: Zebrafish; OL: Medaka; SS: Atlantic salmon; XT: African clawed frog; GG: Chicken; HS: Human.

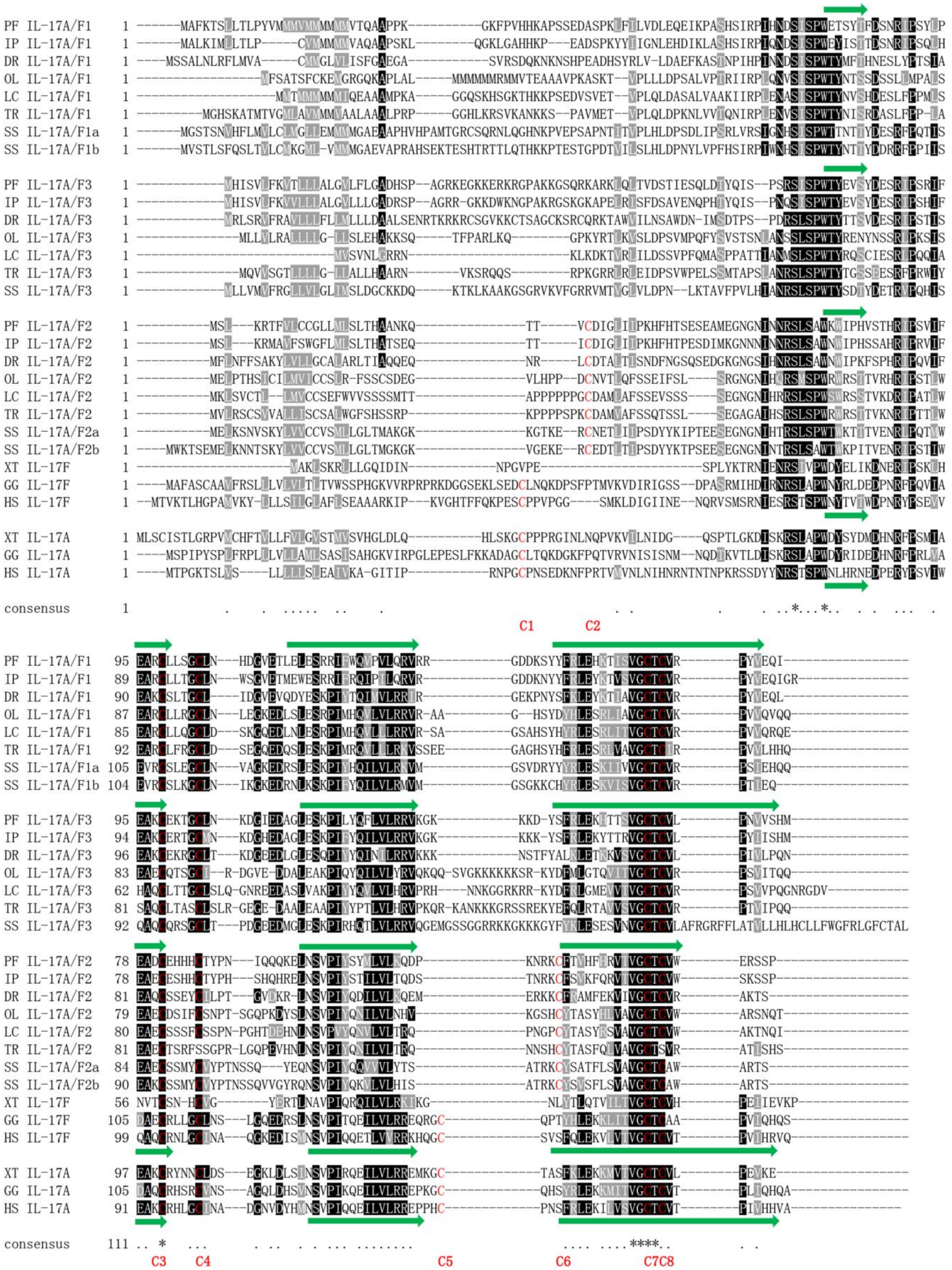
PF IL-17A/F1
IP IL-17A/F1
DR IL-17A/F1
OL IL-17A/F1
LC IL-17A/F1
TR IL-17A/F1
SS IL-17A/F1a
SS IL-17A/F1b
PF IL-17A/F3
IP IL-17A/F3
DR IL-17A/F3
OL IL-17A/F3
LC IL-17A/F3
TR IL-17A/F3
SS IL-17A/F3
PF IL-17A/F2
IP IL-17A/F2
DR IL-17A/F2
OL IL-17A/F2
LC IL-17A/F2
TR IL-17A/F2
SS IL-17A/F2a
SS IL-17A/F2b
XT IL-17F
GG IL-17F
HS IL-17F
XT IL-17A
GG IL-17A
HS IL-17A
consensus
C1
C2
C3
C4
C5
C6
C7C8

Supplemental Table 1. Primers used in this study.

Primer name	Sequence (5'-3')	Ta (℃)	Application
IL-17A/F1-F1	GATGATGATGATGATGATGATGGTC	60	cDNA cloning of IL-17A/F1 gene
IL-17A/F1-R1	TCAGATCTGCTCCACGTACGG		
IL-17A/F1-3'RACE outer	CCACAATGACTCTATCTCACCCTG	60	3'RACE (1st round PCR)
IL-17A/F1-3'RACE inner	TGTTGAGACGCTGGAGTTGG	57	3'RACE (2nd round PCR)
IL-17A/F1-QF	CAGGCTGCTCCTCCAAAG	64	qPCR of IL-17A/F1 mRNA
IL-17A/F1-QR	AGCGTCTCAACACCGTCAT		
Pro-IL-17A/F1-F1	CCGGAATTCATGATGATGATGATGATGATGGTC	58	Prokaryotic expression
Pro-IL-17A/F1-R1	ACGCGTCGACTCAGATCTGCTCCACGTACGG		
IL-17A/F2-F1	ATGAGCTTAAAGCGAACGTTTG	60	cDNA cloning of IL-17A/F2 gene
IL-17A/F2-R1	TCACGGTGACGATCTTTCCC		
IL-17A/F2-3'RACE outer	TTGAGGCAGATTGTGAGCAT	54	3'RACE (1st round PCR)
IL-17A/F2-3'RACE inner	ACCCAAAGAACAGGAAGTGC	55	3'RACE (2nd round PCR)
IL-17A/F2-QF	ATTTGAGGCAGATTGTGAGC	64	qPCR of IL-17A/F2 mRNA
IL-17A/F2-QR	TGTTCTTTGGGTCCTGTTTG		
Pro-IL-17A/F2-F1	CCGGAATTCGCCGCTAACAAACAGACGAC	60	Prokaryotic expression
Pro-IL-17A/F2-R1	ACGCGTCGACTCACGGTGACGATCTTTCCC		
IL-17A/F3-F1	ATGCACATCTCGGTGCTTTTCA	60	cDNA cloning of IL-17A/F3 gene
IL-17A/F3-R1	CTACATGTGAGACACGACGTTCGG		
IL-17A/F3-3'RACE outer	ATTCCGAGCCGCATCTTC	55	3'RACE (1st round PCR)
IL-17A/F3-3'RACE inner	GCTCAGAAGAGTAAAGGGCAAAA	56	3'RACE (2nd round PCR)
IL-17A/F3-QF	ATTGTTGTTGGCATTGGGAGTA	64	qPCR of IL-17A/F3 mRNA
IL-17A/F3-QR	GGTATCTAGTTGGCTTTCTATTGTAG		
Pro-IL-17A/F3-F1	CCGGAATTCGTACTGTTCCTCGGAGCAGATC	60	Prokaryotic expression
Pro-IL-17A/F3-R1	ACGCGTCGACCTACATGTGAGACACGACGTTC		
IL-1β-QF	TCTCAGCCTACAACCCACCA	64	qPCR of IL-1β mRNA
IL-1β-QR	CTCCATTCCATCGTTCTCCT		
TNF-QF	CAGGTTTTGTTGGATGTGGACG	64	qPCR of TNF mRNA
TNF-QR	AGGGAGTGCTTGATTTCTTGTGC		
IFNγ-QF	TATTTGGAATGATGGTGTG	62	qPCR of IFNγ mRNA
IFNγ-QR	GCTTCAAGTTTTCATCCTG		
IL-22-QF	GTGCTGCTTGTATGGTGCTGCT	62	qPCR of IL-22 mRNA
IL-22-QR	TGTTGTTCCAGGTGTCAGAGTTGTC		
IL-6-QF	CACTATCTTGCCCTGTTCCTG	62	qPCR of IL-6 mRNA
IL-6-QR	TCGTGTTCTGTGTTCCTCCG		

Primer name	Sequence (5'-3')	Ta (℃)	Application
IL-11-QF	GCTCTTCTCCGTCCTATTGG	62	qPCR of IL-11 mRNA
IL-11-QR	AGGTCGTGGGTCATCTTCTG		
CXCL1-QF	GTCCTGACCTTCATCACCTT	62	qPCR of CXCL1 mRNA
CXCL1-QR	TCAATCACTTTCATTACCCA		
CXCL8-QF	CAGTGTTGTTCGTCATCATTTTTG	64	qPCR of CXCL8 mRNA
CXCL8-QR	TGGATTCAGGCAGACCTTCATT		
CXCL11-QF	TCACAGTGTTGTTCGTCATC	62	qPCR of CXCL11 mRNA
CXCL11-QR	TCATTCCTTGCTTCAGAGTTA		
CCL3-QF	CTTTCAGACACGACCAATC	62	qPCR of CCL3 mRNA
CCL3-QR	TAAACAGACGCTCATCAAT		
CCL4-QF	CTCTGCTCTCTTCATCTGGT	62	qPCR of CCL4 mRNA
CCL4-QR	GAGTTGGGGTGTTCCTTC		
IL-17RA-QF	AAAGCCCATCAGGCAAAG	62	qPCR of IL-17RA mRNA
IL-17RA-QR	TCTGGTCCGCACGGTGTA		
ACT1-QF	GAACAAGTGGATGGACGG	62	qPCR of ACT1 mRNA
ACT1-QR	GGGAAGAGAACAGGGACG		
TRAF2-QF	GTGGACATCGTTTCTGCGT	62	qPCR of TRAF2 mRNA
TRAF2-QR	TGCTGACCCTCGTAATCTTTC		
TRAF5-QF	AAACGAAGCAAAGTGAAAGTC	62	qPCR of TRAF5 mRNA
TRAF5-QR	CTCAGCATCCAGAACATTACG		
TRAF6-QF	TTCGGGGTCAGTTGTCGT	62	qPCR of TRAF6 mRNA
TRAF6-QR	ATGTTCGGATGGGGGTG		
TAK1-QF	AATCAGAAAGGAATGCG	62	qPCR of TAK1 mRNA
TAK1-QR	GCCGTGTAGTGGGGTAG		
S100A1-QF	GGGCAATAGCACCACAC	62	qPCR of S100A1 mRNA
S100A1-QR	CCACCACAAGGGACACA		
LEAP-QF	TGGCGCTGGAACACATTGA	62	qPCR of LEAP mRNA
LEAP-QR	GGACGAAGAGGATTTAGGCTGG		
β-defensins-QF	CAATGGCAGCATTTCCCTGGAGTT	64	qPCR of β-defensins mRNA
β-defensins-QR	CGTGAGACACACAGCAAACAAACC		
Oligo(dT)$_{17}$	GACTCGAGTCGACATCGA (T)$_{17}$		Universal primer for 3'RACE
Linker adapter	GACTCGAGTCGACATCG		
β-actin-QF	TCCCTGTATGCCTCTGGTCGT	64	qPCR of β-actin mRNA
β-actin-QR	AAGCTGTAGCCTCTCTCGGTC		

Ta: annealing temperature; RACE: rapid amplification of cDNA ends.

Supplemental Table 2. Amino acid sequence homology of *Pf*_IL-17A/Fs with other known sequences.

Species	Molecule	Similarity (%) of *Pf*_IL-17A/F			Identity (%) of *Pf*_IL-17A/F		
		IL-17A/F1	IL-17A/F2	IL-17A/F3	IL-17A/F1	IL-17A/F2	IL-17A/F3
Channel catfish	IL-17A/F1	69.2	27.2	39.8	65.4	19.8	30.7
Zebrafish		51.0	25.3	44.0	37.6	17.9	33.7
Medaka		44.3	24.8	42.2	35.3	15.5	30.7
Channel catfish	IL-17A/F2	27.4	79.1	30.4	20.4	74.1	21.1
Zebrafish		26.3	55.6	30.7	20.0	47.2	21.7
Medaka		30.4	41.3	29.5	20.5	32.7	18.7
Channel catfish	IL-17A/F3	39.0	33.8	83.8	28.7	21.9	77.5
Zebrafish		38.6	30.4	57.4	27.7	24.4	46.3
Medaka		36.1	27.8	42.8	26.0	19.5	32.4
African clawed frog	IL-17A	34.3	29.3	34.5	23.7	21.0	24.6
Chicken		30.7	29.7	35.1	19.3	17.7	24.1
Human		30.2	28.3	37.7	23.5	21.4	30.5
African clawed frog	IL-17F	27.3	28.8	30.7	18.8	20.9	22.7
Chicken		34.1	28.0	32.2	24.4	18.3	23.6
Human		28.7	27.2	37.8	20.1	20.8	25.0
